# Identification of ABC Transporter Genes of *Fusarium graminearum* with Roles in Azole Tolerance and/or Virulence

**DOI:** 10.1371/journal.pone.0079042

**Published:** 2013-11-11

**Authors:** Ghada Abou Ammar, Reno Tryono, Katharina Döll, Petr Karlovsky, Holger B. Deising, Stefan G. R. Wirsel

**Affiliations:** 1 Institute of Agricultural and Nutritional Sciences, Faculty of Natural Sciences III, Martin-Luther-University Halle-Wittenberg, Halle (Saale), Germany; 2 Molecular Phytopathology and Mycotoxin Research Section, Georg-August-Universität Göttingen, Göttingen, Germany; 3 Interdisziplinäres Zentrum für Nutzpflanzenforschung, Martin-Luther-Universität Halle-Wittenberg, Halle (Saale), Germany; University of Wisconsin - Madison, United States of America

## Abstract

*Fusarium graminearum* is a plant pathogen infecting several important cereals, resulting in substantial yield losses and mycotoxin contamination of the grain. Triazole fungicides are used to control diseases caused by this fungus on a worldwide scale. Our previous microarray study indicated that 15 ABC transporter genes were transcriptionally upregulated in response to tebuconazole treatment. Here, we deleted four ABC transporter genes in two genetic backgrounds of *F. graminearum* representing the DON (deoxynivalenol) and the NIV (nivalenol) trichothecene chemotypes. Deletion of *FgABC3* and *FgABC4* belonging to group I of ABC-G and to group V of ABC-C subfamilies of ABC transporters, respectively, considerably increased the sensitivity to the class I sterol biosynthesis inhibitors triazoles and fenarimol. Such effects were specific since they did not occur with any other fungicide class tested. Assessing the contribution of the four ABC transporters to virulence of *F. graminearum* revealed that, irrespective of their chemotypes, deletion mutants of *FgABC1* (ABC-C subfamily group V) and *FgABC3* were impeded in virulence on wheat, barley and maize. Phylogenetic context and analyses of mycotoxin production suggests that *FgABC3* may encode a transporter protecting the fungus from host-derived antifungal molecules. In contrast, *FgABC1* may encode a transporter responsible for the secretion of fungal secondary metabolites alleviating defence of the host. Our results show that ABC transporters play important and diverse roles in both fungicide resistance and pathogenesis of *F. graminearum*.

## Introduction

*Fusarium* head blight (FHB), caused by a number of closely related species including *Fusarium graminearum* Schwabe (teleomorph *Gibberella zeae* (Schwein.) Petch), is a major disease of wheat and other small-grain cereals. These fungi can cause considerable economic losses not only due to diminishing yield and quality of the harvest but also because of the production of mycotoxins in infected grains [1]. In *F. graminearum*, the most important mycotoxins are B-trichothecenes such as deoxynivalenol (DON) and nivalenol (NIV), but also zearalenone (ZEN) [1], [2]. Infection of cereals leading to contamination of food and feed with these mycotoxins poses a health risk to consumers. The major sources of inoculum in FHB are ascospores produced by *F. graminearum* growing saprophytically on cereal debris. After expulsion from the perithecium, airborne ascospores infect wheat heads. Infection occurs most effectively at the stage of anthesis. Some FHB-causing fungi including *F. graminearum* may infect cereals at other developmental stages resulting in seedling blight, foot, crown or root rots [1].

Control of FHB includes agronomic practices such as appropriate crop rotation, tilling and fungicide application, and the utilisation of resistant cultivars. Management practices integrating several control measures performed better than the application of measures separately [3], [4]. In North America and Europe, the preferred fungicides to control FHB are triazoles such as tebuconazole, prothioconazole and metconazole, all of which are sterol biosynthesis inhibitors (SBI) class I [5]. Recently, declining efficacies of these fungicides was reported [6], [7].

In our previous work, we investigated the capability of *F. graminearum* to develop resistance to azoles and the molecular mechanisms underlying this process. Cultivation of strain NRRL 13383 in the presence of a sublethal concentration of tebuconazole allowed to recover isolates with enhanced tolerance to that fungicide [8]. Transcriptome analysis of *F. graminearum* challenged with tebuconazole *in vitro* [9] showed strong responses for some genes of the sterol biosynthesis pathway, noticeably *FgCyp51A* to *FgCyp51C* encoding cytochrome P450 sterol 14α-demethylase, which is the molecular target of azoles. Furthermore, 15 out of 54 genes encoding ABC transporters were more than twofold upregulated by tebuconazole treatment. Functional proof for a contribution of CYP51 to azole resistance in *F. graminearum* was provided by deletion analyses [10], [11]. It is however uncertain whether mutations in any of the three *Cyp51* genes or changes in their regulation cause increased azole tolerance in field strains.

In addition to CYP51, membrane-bound transporters affect the sensitivity of fungal pathogens to azoles [12], [13], [14]. Contribution of these proteins to azole resistance in *F. graminearum* has not been shown before. Taking advantage of our previous transcriptome analysis, we have chosen in this study four genes encoding ABC transporters for functional analyses. We deleted these genes to determine their contribution to fungicide resistance, virulence and mycotoxin production.

## Materials and Methods

### Fungal Cultivation

The strains *F. graminearum* PH1 and NRRL 13383 used in this study, as well as the procedures used for their growth, sporulation and storage were described previously [8], [9].

Vegetative growth rates were determined on PDA plates (Ø 90 mm) at 15°C, 23°C and 30°C. Mycelial plugs (Ø 5 mm) taken from margins of colonies grown on PDA at 23°C for five days were used for inoculation. Two perpendicular measurements of colony diameters were taken during seven days and averaged. Each variant was replicated four times.

The capacity of fungal strains to produce macroconidia was determined in 50 ml Mung Bean Broth (MBB) [15] in 250 ml Erlenmeyer flasks inoculated with five mycelial plugs per flask as above. Cultures were incubated at 23°C with 100 rpm for 7 days. Conidia were harvested by filtering through Miracloth (Merck, Darmstadt, Germany) and collected by centrifugation at 3000×g for 10 min. Conidial density was determined using a haemacytometer (Brand, Wertheim, Germany). Each strain was grown in four cultures and conidia were counted twice. Statistical significances were determined by T-test (*p*<0.05) as implemented in the Microsoft Excel 2010 software.

Germination efficiency of macroconidia was determined on glass slides inoculated with 20 µl of a conidial suspension (10^4^ ml^−1^), covered with a cover glass and incubated on three layers of moistened paper towels inside a 120×120×17 mm plastic dish (Greiner Bio-One, Solingen, Germany) at 23°C for 24 h under illumination with near-UV light (L18W/73, Osram, Munich, Germany). Germinated and ungerminated conidia were counted twice in four replicates per strain. Statistical analysis was carried out as described above.

### Procedures for Nucleic Acid Isolation Manipulations

The isolation of fungal genomic DNA and total RNA followed published methods [9]. Cultivation and treatment of mycelia with tebuconazole, preparation and validation of RNA, RT-qPCR and data analysis were performed as outlined previously [9]. Each variant was represented by four biological replicas (RNAs from independent cultures), each of which was analysed twice by RT-qPCR. Expression data of candidate genes were normalised with those from three reference genes (FGSG_01244, FGSG_06245, FGSG_10791) as reported [9].

DNA fragments used for fungal transformation were generated by the DJ-PCR method [16]. Marker genes employed for selection encoded hygromycin phosphotransferase (*hph*), nourseothricin acetyltransferase (*nat1*) and neomycin phosphotransferase (*npt*). Using plasmids pAN7-1 [17], pNR1 [18] and pII99 [19] as templates, DNA fragments comprising these markers genes including heterologous constitutive promoters were generated by PCR and then fused by DJ-PCR with the left and right flanks of the respective target gene. The deletion constructs generated included *hph* for *FgABC1* (FGSG_10995), *nat1* for *FgABC2* (FGSG_17046) and *FgABC4* (FGSG_17058) and *npt* for *FgABC3* (FGSG_04580) (see Fig. S1). Oligonucleotides used in this study are listed in Table S1.

### Generation and Validation of *F. graminearum* Transformants

For the preparation of protoplasts, 5×10^6^ macroconidia were incubated for 12 h in 100 mL of YEPD at 28°C and 175 rpm. The mycelium was recovered on a sterile paper filter and then incubated for 4 h at 30°C and 90 rpm in 20 ml of protoplasting mix (500 mg driselase, 1 mg chitinase, 100 mg lysing enzyme of *Trichoderma harzianum* (all from Sigma-Aldrich, Schnelldorf, Germany) in 1.2 M KCl). Protoplasts were harvested at R.T. by centrifugation at 1000×g and suspended in 1 mL STC buffer (1.2 M sorbitol, 50 mM CaCl_2_, 10 mM Tris-HCl, pH 7.5). A transformation reaction contained 10^7^ protoplasts in 100 µL STC buffer, 50 µL 30% PEG 8000 and 8 µg DNA of the deletion construct in 50 µL water. After incubation for 20 min at RT and 50 rpm, 2 mL 30% PEG 8000 and 5 min later 4 mL STC buffer were added. Aliquots of 600 µL were mixed with 15 mL molten regeneration medium (275 g of sucrose, 0.5 g yeast extract, 0.5 g casein hydrolysate, 5 g of agar per litre) and poured into a Petri dish. After incubation for 12 h at 26°C, 15 mL of molten regeneration medium containing, depending on the marker gene used for selection, either hygromycin B, nourseothricin or G418 at concentrations of 200 µg/mL, were poured onto the surface of the agar. Colonies that started emerging after 4 d were harvested to obtain single spore isolates as described before [8].

Transformants were analysed by PCR and Southern hybridisation. Probes for the latter were generated with the PCR DIG Labeling Mix^PLUS^ kit (Roche Diagnostics, Mannheim, Germany) as recommended by the manufacturer. Bioluminescence was visualized by exposing Nylon membranes to Hyperfilm ECL X-ray film (Amersham Pharmacia Biotech, Piscataway, USA).

### Determination of Sensitivity to Fungicides and Plant Metabolites

The sensitivity of transformants to fungicides and plant compounds was tested on PDA plates (12×12 cm, Greiner Bio-One) amended with appropriate concentrations of a given substance. For each compound, we used three concentrations that were optimized in preliminary experiments. The following fungicides were obtained as commercial formulations: azoxystrobin (Amistra, BASF), fenpropimorph (Corbel, BASF), metconazole (Caramba, BASF), prochloraz (Sportak, BASF) and tebuconazole (Folicur, Bayer). Pure active compounds epoxyconazole, fenarimol, spiroxamine, boscalid and dithianon were obtained from Sigma-Aldrich (Schnelldorf, Germany), except for prothioconazole, which was kindly provided by Bayer. Tolnaftat (Sigma-Aldrich) was included as a control xenobiotic that has never been used in agriculture. Sensitivity of fungal strains against plant secondary metabolites 2-benzoxazolinone ( = BOA), 3-(dimethylaminomethyl)indole ( = gramine), 2,3-dihydro-5,7-dihydroxy-2-(4-hydroxyphenyl)-4H-1-benzopyran-4-one, 4′,5,7-trihydroxyflavanone ( = naringenin) and 3,3′,4′,5,7-pentahydroxyflavone dihydrate ( = quercetin) purchased from Sigma-Aldrich was tested in the same way. For all substances, stock solutions were prepared in DMSO; the final concentration of the solvent in culture media was at most 0.3%. Two µL of a suspension of macroconidia (1×10^5^ mL^−1^) were used as inoculum to assess the effect of the compounds on germination. Mycelial plugs on unamended PDA were used as inoculum to determine the effect of the compounds on vegetative growth. At least three plates were used for each compound and concentration. Incubation was carried out in the dark at 23°C for strains derived from NRRL 13383 and at 30°C for strains with PH-1 background. Colony area was determined on digital photographs using ImageJ software version 1.46 (http://rsbweb.nih.gov/ij/index.html). Statistical analysis was done as above.

### Wheat Ear Infection Assay

Cultivation of wheat in the greenhouse and environmentally controlled growth chambers and ear inoculation was described earlier [8]. Shortly, *F. graminearum* strains were point-inoculated into the 9^th^ spikelet of wheat cultivar Kadrilj (SW Seed Hadmersleben, Hadmersleben, Germany) when it reached anthesis. The inoculum consisted of 300 macroconidia suspended in 10 µL of 0.02% Tween 20. For each strain tested, at least ten wheat heads were inoculated and covered with plastic bags misted with water to maintain high humidity. The bags were removed after 2 days post inoculation (dpi) and the incubation continued at 25°C, 70% relative humidity until 14 dpi. The development of bleached spikelets in the heads was recorded daily.

### Maize Stem Infection Assay

Maize plants cultivar Golden Jubilee (Territorial Seed Company, Cottage Grove, OR, USA) were cultivated for six weeks in a greenhouse at 24°C with 50% relative humidity and a 14 h photoperiod, which employed lamps (Plantstar 600 Watt E40, Osram, Munich, Germany) providing 4.2×10^17^ photons sec^−1^ m^−2^ at the surface of the bench. For each strain tested, at least five plants were inoculated by punching a hole into the stem at the first internode using a sterile tootpick, followed by injection of 1000 macroconidia in 10 µL of 0.02% Tween 20. The control plants were inoculated with 0.02% Tween 20. The hole was covered with Parafilm for 7d to maintain high humidity and exclude other organisms. At 14 dpi, the stalks were split longitudinally and the symptoms were documented by photography. The extent of the necrotic area was quantified using ImageJ software version 1.46 as above. Statistical analysis was performed as above.

### Barley Ear Infection Assay

Barley cultivar Barke (Saatzucht Josef Breun, Herzogenaurach, Germany) was cultivated for ten weeks in a greenhouse using the same conditions as described for maize. For each strain tested, sixteen mature ears were inoculated employing a glass flacon to spray 2000 macroconidia in 2 ml of 0.02% Tween 20 onto each ear. The inoculated ears were enclosed in a misted plastic bag for 2d. After an additional incubation of 12d, the number of bleached spikelets was recorded for each head. Statistical analysis was carried out as above.

### Analysis of Mycotoxin Production *in vitro*

*Fusarium* isolates were grown in rice media, culture material was extracted with acetonitrile/water and the extracts were defatted as described [20]. Mycotoxins were separated by HPLC on an RP column (Polaris C18 ether, 100×2 mm, 3 µm particle size; Agilent, Darmstadt, Germany) at 40°C at a flow rate of 0.2 ml/min. The solvent system consisted of (A) water with 5% acetonitrile and (B) methanol, both containing 7 mM acetic acid. The elution gradient rose linearly from 10% to 98% of solvent B followed by washing and equilibration steps. The detection was performed by tandem mass spectrometry using triple quadrupole 1200L (Varian, Darmstadt, Germany) after electrospray ionization in negative mode as described before [21], [22]. For each isolate, at least five independent cultures were analysed. Statistical analysis was done as above.

## Results

### Targeted Deletion of ABC Transporter Genes *FgABC1*, *FgABC2*, *FgABC3* and *FgABC4*

We chose four genes encoding ABC transporters, which were previously found upregulated in strain PH-1 after tebuconazole treatment, for targeted deletion mutagenesis. These genes clustered in three ABC subfamilies [9]. ABC transporters have been classified according to several schemes. Using one that was based on the yeast nomenclature classified FgABC1 (FGSG_10995) and FgABC4 (FGSG_17058) into the MRP (multidrug resistance-related protein) subfamily, FgABC3 (FGSG_04580) into the PDR (pleiotropic drug resistance) subfamily and FgABC2 (FGSG_17046) into a distinct unnamed clade [9]. Applying an alternative classification scheme, which was originally used to classify human ABC transporters, FgABC1 and FgABC4 were assigned to subfamily ABC-C group V, FgABC3 to subfamily ABC-G group I and FgABC2 to subfamily ABC-A group I [23]. Since expression of these four genes was previously only analysed in strain PH-1, we determined their transcript levels by RT-qPCR in strain NRRL 13383 after a 12 h treatment with 5 ppm tebuconazole. In comparison to untreated controls, the transcript levels of *FgABC1*, *FgABC2*, *FgABC3* and *FgABC4* were increased 3.0-, 3.1-, 3.4- and 3.9-fold, respectively. The transcriptional responses of strain NRRL 13383 were thus similar to those previously observed in strain PH-1 [9], except for gene *FgABC4*, which responded stronger in PH-1 (10.6-fold). We introduced deletions of these four genes into both strains, i.e. NRRL 13383 and PH-1, which have the NIV and the 15ADON chemotypes, to examine whether any of the resulting phenotypes may occur independently from the genetic background of the host used for transformation including its chemotype.

DNA cassettes, which comprised a dominant resistance marker gene for an antibiotic controlled by a heterologous promoter and the left and right flanks of the targeted genes, were transformed into both strains, i.e. PH-1 and NRRL 13383 (Fig. S1). Single spore isolates were analysed by PCR and Southern hybridisation for the mode of DNA integration (Fig. S1). Several transformants containing the resistance cassette integrated by a double-cross-over event at the targeted locus (type III integration according to [24]) were obtained for each gene and recipient strain.

### Vegetative Fitness and Fungicide Sensitivities of the Deletion Mutants

We assessed whether the deletion of a given ABC transporter gene would impair the resulting transformants with respect to vegetative growth and asexual reproduction. For each deletion, we examined growth rates on PDA at three temperatures (Fig. S2) and quantified the formation (Fig. S3) and the germination of macroconidia *in vitro* in two transformants (Fig. S4). None of the deletions led to any significant change in any of the three attributes. This was true for the transformants in the PH-1 and the NRRL 13383 backgrounds.

**Figure 1:**
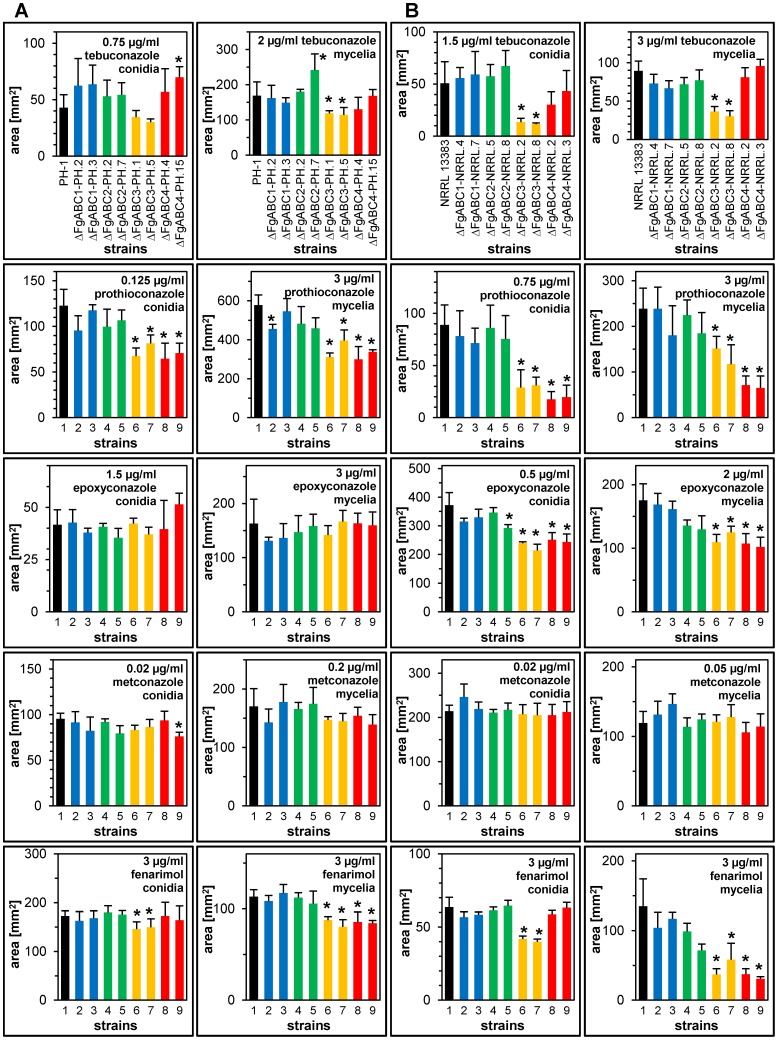
Sensitivity to SBI class I fungicides. For each deletion, colonial areas of two transformants of each genetic background were assessed on PDA amended with the indicated concentration of fungicides. Error bars represent SD. An asterisk indicates a significant difference between a mutant and the wild type. (A) PH-1 background, strains tested: 1) PH-1, 2) ΔFgABC1-PH.2, 3) ΔFgABC1-PH.3, 4) ΔFgABC2-PH.2, 5) ΔFgABC2-PH.7,6) ΔFgABC3-PH.1, 7) ΔFgABC3-PH.5, 8) ΔFgABC4-PH.4, 9) ΔFgABC4-PH.15; (B) NRRL 13383 background, strains tested: 1) NRRL 13383, 2) ΔFgABC1-NRRL.4, 3) ΔFgABC1-NRRL.7, 4) ΔFgABC2-NRRL.5, 5) ΔFgABC2-NRRL.8, 6) ΔFgABC3-NRRL.2, 7) ΔFgABC3-NRRL.8, 8) ΔFgABC4-NRRL.2, 9) ΔFgABC4-NRRL.3.

We determined the impact of 11 fungicides belonging to the chemical groups of anthraquinones, imidazoles, methoxy-acrylates, morpholines, pyridine-carboxamides, pyrimidines, spiroketal-amines and triazoles on germination and vegetative growth of the transformants. In the background of NRRL 13383, we observed significantly reduced tolerance in Δ*FgABC3* strains for the triazoles tebuconazole, prothioconazole and epoxyconazole (Fig. 1). Similarly, Δ*FgABC4* mutants were significantly less tolerant for the latter two. In addition, both of these deletions led to significantly reduced tolerance against fenarimol, which has the same target as the triazoles (SBI class I) but is a pyrimidine. There existed no significantly changed sensitivities against fungicides grouped into SBI class II, QoI, SDHI, anthraquinone and N-phenyl carbamate, as well as tolnaftat. The deletion of the same four ABC transporter genes in the PH-1 background affected the resulting mutants in a similar way as in NRRL 13383. However, the reductions in tolerance to the above-mentioned fungicides were less severe so that in some cases these trends were not statistically significant (Fig. 1). Deletion of the genes *FgABC1* and *FgABC2* did not significantly reduce the tolerance levels for any fungicide in any of the two backgrounds. The impact of the SBI class I fungicides prothioconazole and fenarimol on vegetative hyphae was observed by microscopy (Fig. S5). In untreated control cultures, hyphal morphology of all mutants resembled that of the wild type strains. In contrast, treatment with 3 ppm of either fungicide induced aberrant hyphal morphology in Δ*FgABC3* and Δ*FgABC4* mutants, but not in the WT and in Δ*FgABC1* and Δ*FgABC2* mutants. Such hyphae appeared thicker and had swellings that emerged throughout the mycelium, but most often apically. Occasionally, such structures collapsed. These effects resembled those reported previously for tebuconazole treatment of *Fusarium culmorum* [25].

We examined whether the deletion of the four genes encoding ABC transporters might have affected the sensitivity of the transformants to four commercially available secondary metabolites with antifungal properties produced by cereals. However, at none of the concentrations tested neither BOA, gramine, naringenin nor quercetin impaired the growth of any mutant in any background significantly different from the respective wild type strain (not shown).

### Transcript Abundances of *FgABC1* to *FgABC4* in the Deletion Mutants

**Figure 2:**
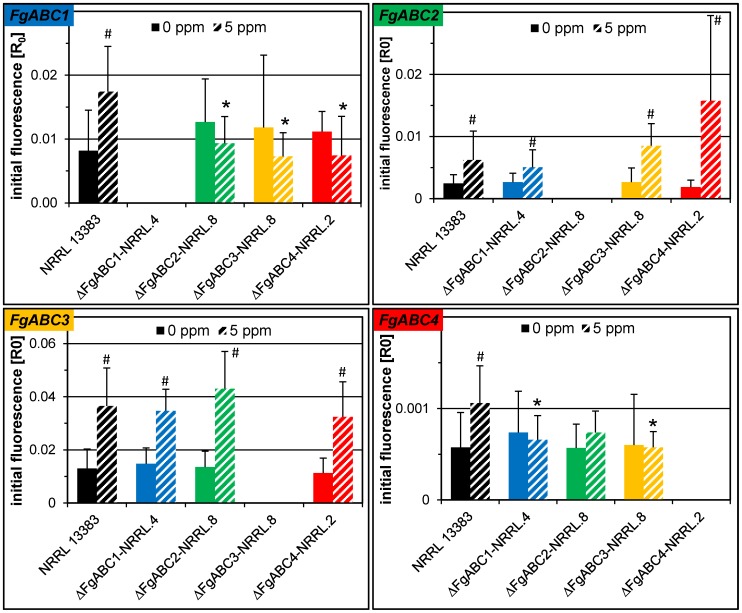
Transcript levels determined by RT-qPCR. For each deletion, one transformant of the NRRL 13383 background was analysed. Columns show calculated initial fluorescence after normalisation with three reference genes. The analysed gene is indicated in the upper left corner of a given box. For each strain, RNA preparations were assayed originating from cultures amended with 5# indicate significant differences between the fungicide treatment and the control. Within each treatment, an asterisk indicates a significant difference between a mutant and the wild type.

We determined by RT-qPCR the transcript levels of the four ABC transporter genes in untreated and tebuconazole-treated wild type NRRL 13383 and a single mutant for each deletion. As expected, no transcripts were detected for the deleted gene in the mutant that was deleted for that gene (Fig. 2). In the wild type, the transcript levels of all genes increased significantly after tebuconazole treatment (Fig. 2, #). Interestingly, this transcriptional response was lost for *FgABC1* in the deletion mutants of genes *FgABC2*, *FgABC3* and *FgABC4*. Likewise, transcriptional responses to the fungicide treatment were also lost for *FgABC4* in the deletion mutants of genes *FgABC1*, *FgABC2* and *FgABC3*. The same two genes, i.e. *FgABC1* and *FgABC4*, were the only showing significant differences when comparing the mutants to the wild type (Fig. 2, asterisks). Transcript levels of *FgABC1* were significantly lower in the deletion mutants of genes *FgABC2*, *FgABC3* and *FgABC4*, but only in cultures treated with tebuconazole. The corresponding effects were observed for gene *FgABC4*, although this proved only significant for the deletion mutants of *FgABC1* and *FgABC3*.

### Virulence of the Deletion Mutants

**Figure 3:**
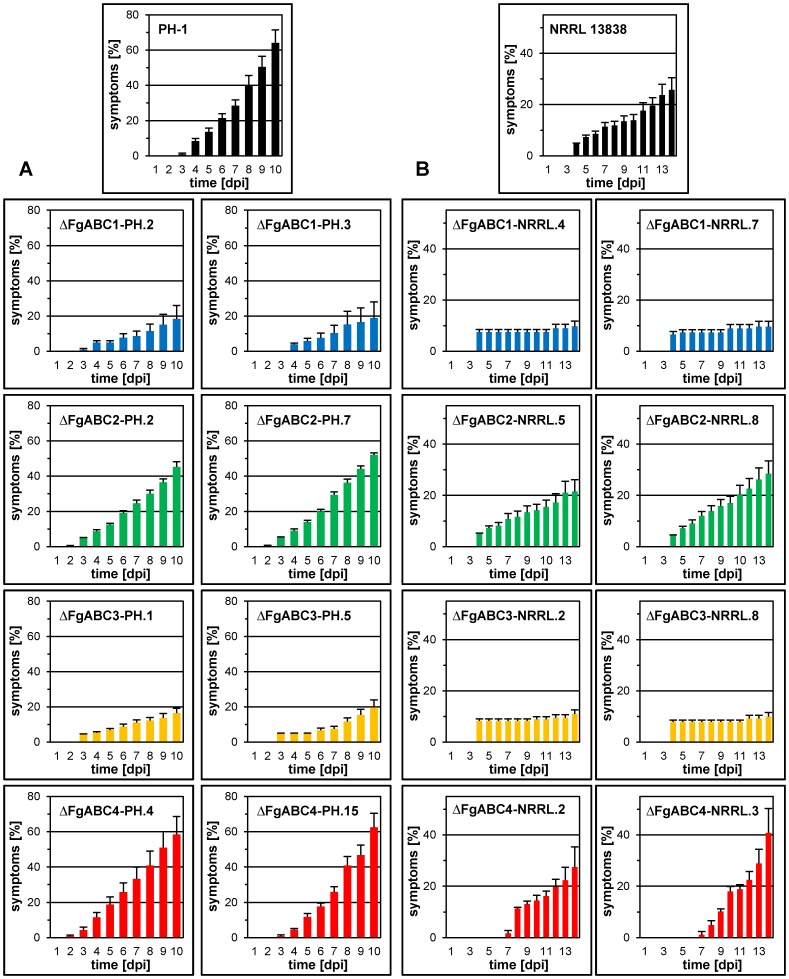
Virulence on wheat heads. For each deletion, symptom development of two transformants of each genetic background is compared to the respective wild type strain for up to 14-inoculated wheat heads. Error bars represent SE. A) PH-1 background, B) NRRL 13383 background.

**Figure 4:**
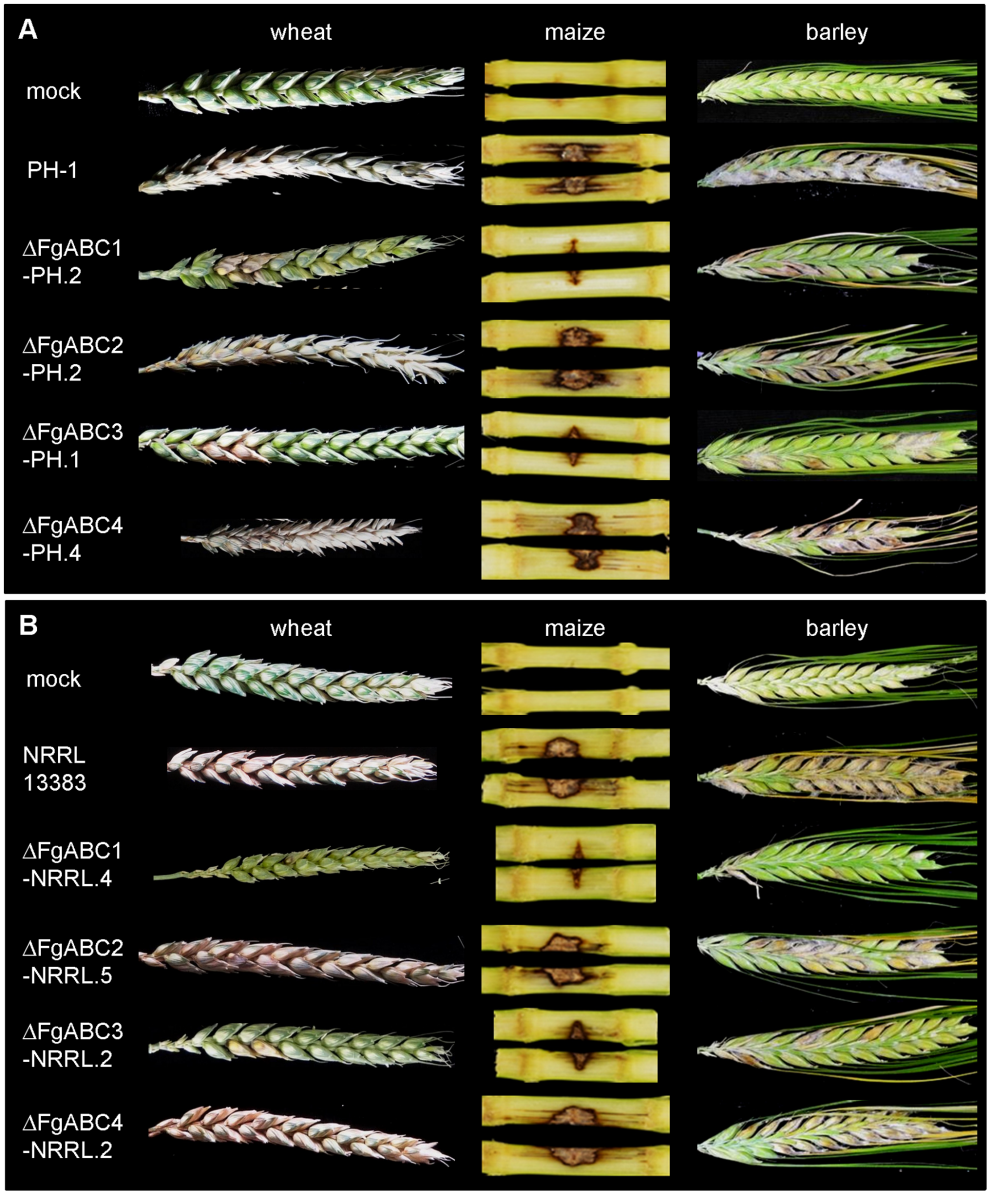
Symptoms on infected cereals. Photos show symptoms occurring at the end of the monitoring. One representative example is provided for each genotype. Mocks were treated with 0.02% Tween 20. A) PH-1 background, B) NRRL 13383 background.

For each background and each deleted gene, two transformants were point-inoculated into central spikelets of wheat ears. Over the entire period monitored, the percentage of bleached spikelets per head was higher in heads inoculated with strain PH-1 than with strain NRRL 13383 (Figs. 3, 4). Deletion of *FgABC1* caused a strong reduction of virulence in both backgrounds. Although the mutants were still able to cause local infections, they spread very slowly, as compared to the corresponding wild type strains. At the end of the scoring period, in the PH-1 background the Δ*FgABC1* mutants had caused disease in only about one third of the spikelets, as compared to wild type strain (Fig. 3a). In the NRRL 13383 background, symptom development was even slower (Fig. 3b). Deletion of *FgABC3* also resulted in strongly reduced symptoms in both genetic backgrounds, comparable to Δ*FgABC1* deletion strains. In contrast, deletion mutants of *FgABC2* and *FgABC4* caused symptom developments resembling those of the corresponding wild type references (Fig. 3).

**Figure 5:**
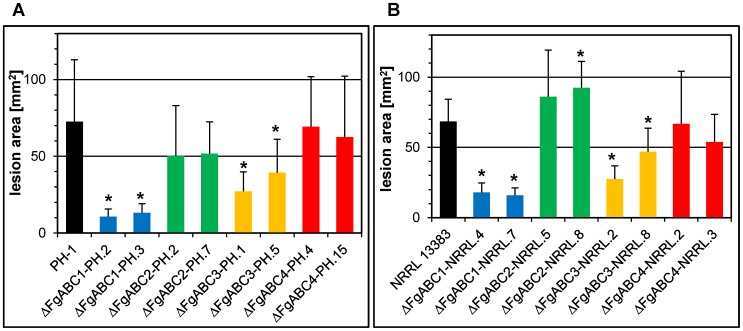
Virulence on maize stems. For each deletion, two transformants of each genetic background are compared to the respective wild type strain. Columns give symptomatic areas in maize stems that were harvested at 14) PH-1 background, B) NRRL 13383 background.

**Figure 6:**
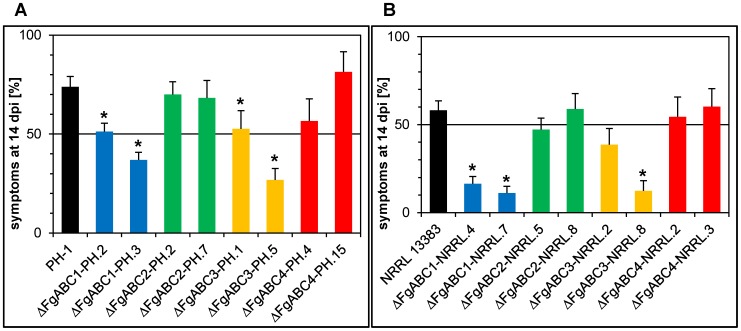
Virulence on barley heads. For each deletion, two transformants of each genetic background are compared to the respective wild type strain. Columns show the percentage of symptomatic spikelets in spray-inoculated barley heads at 14 dpi. Error bars represent SE. A) PH-1 background, B) NRRL 13383 background.

*F. graminearum* has a rather broad host range encompassing several cultivated and wild grasses, allowing to determine whether virulence factors discovered to be essential for infection of wheat are also essential for the infection of other host species. Interestingly, the same ABC transporter genes required for full virulence in wheat were also required for full virulence in maize (Figs. 4, 5) and barley (Figs. 4, 6). Compared to the respective wild type strains, deletion mutants of *FgABC1* and *FgABC3* were significantly reduced for virulence on maize stems, which was true in both genetic backgrounds, i.e. PH-1 (Fig. 5a) and NRRL 13383 (Fig. 5b). Virulence defects were more severe for Δ*FgABC1* than for Δ*FgABC3* strains. In barley, the reduction in virulence of the Δ*FgABC1* mutants was more evident in the NRRL 13383 (Fig. 6b) than in the PH-1 background (Fig. 6a). The Δ*FgABC3* mutants showed similar effects in both backgrounds.

### Production of Mycotoxins by the Deletion Mutants

**Figure 7:**
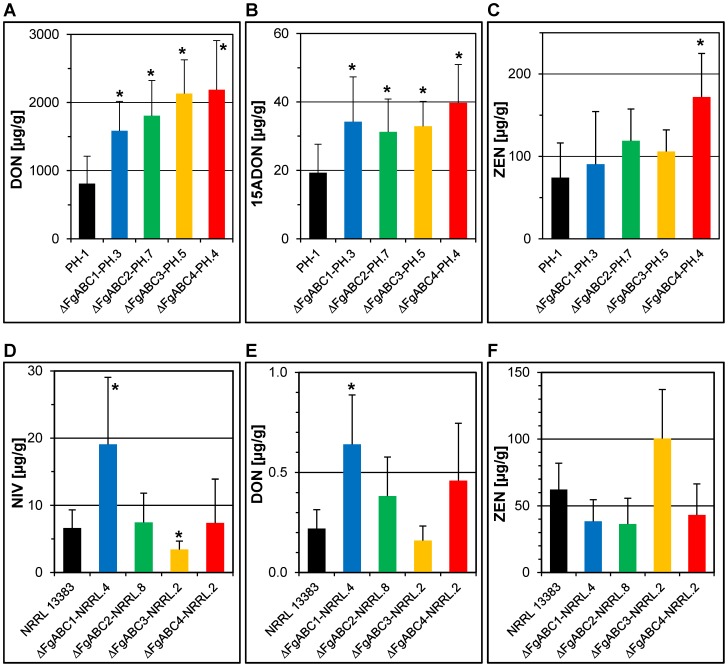
Mycotoxin production. For each deletion, one transformant of each genetic background is compared to the respective wild type strain. Columns show the concentration of a given substance in rice cultures determined by HPLC-MS/MS. Error bars represent SD. An asterisk indicates a significant difference between a mutant and the wild type. A) to C) PH-1 background, D) to F) NRRL 13383 background. A), E) DON; B) 15ADON; C), F) ZEN; D) NIV.

We analysed whether the deletion of the ABC transporter genes affected the levels of B-trichothecenes and zearalenone, and whether possible alterations might explain the results seen in the virulence assays. In the PH-1 background, the levels of DON (Fig. 7a) and 15ADON (Fig. 7b) produced *in vitro* were increased in all four deletion mutants when compared to the wild type strain. The ZEN levels produced by the mutants were similar to PH-1, except for the deletion mutant of *FgABC4* that showed higher levels (Fig. 7c). In the NRRL 13383 background, deletion of *FgABC1* led to higher and deletion of *FgABC3* led to lower NIV levels, whereas the other two mutants resembled the wild type (Fig. 7d). None of the deletion mutants produced ZEN (Fig. 7f) at levels that differed significantly from the wild type. This experiment indicated that the strongly reduced virulence observed in the deletion mutants of *FgABC1* and *FgABC3* was likely not caused by a reduction of trichothecenes that represent virulence factors for the infection of wheat and maize, but not barley [26].

## Discussion

We study mechanisms mediating azole resistance in *F. graminearum*. Exploiting transcriptomic and phylogenetic data [9], we chose four genes for functional analyses encoding full-size ABC transporters belonging to three subfamilies. We found that the deletion mutants Δ*FgABC3* and Δ*FgABC4* had acquired a higher sensitivity to several fungicides belonging to the SBI class I. Remarkably, the deletion of *FgABC1* and *FgABC3* caused a strong reduction of virulence on three economically important crops, wheat, barley and maize.

### Effects of Deletions on Fungicide Sensitivity

Our study shows that the deletion of *FgABC3* and of *FgABC4* caused enhanced sensitivity to several triazoles and to fenarimol that are classified as SBI class I fungicides. These effects are rather specific since such deletion mutants do neither suffer from general fitness impairment nor do they show increased sensitivity against other fungicide classes. Considering that the genome of *F. graminearum* comprises 54 genes putatively encoding ABC transporters [9], it is notable that single deletions already yielded this phenotype. In addition to *FgABC3* and *FgABC4*, the PDR and the MRP subfamilies harbour additional genes that also responded to tebuconazole [9]. This could suggest that distinct transcriptional responses only occurring for *FgABC3* and *FgABC4* may not explain why similar paralogs were not able to complement the deletions. Nonetheless, our RT-qPCR analyses revealed for two of the four genes, *FgABC1* and *FgABC4*, a loss of transcriptional upregulation, which occurs in the wild type, in response to triazole, if any of the other three genes studied was deleted. In triazole-treated Δ*FgABC3* mutants, transcript levels of *FgABC4* were significantly lower than those of the fungicide-treated wild type. As shown above, *FgABC4* is needed to maintain wild type levels of fungicide tolerance. Thus, the reduced sensitivity in the Δ*FgABC3* mutants might have been indirectly caused by the decrease of *FgABC4* transcript levels. However, this is unlikely because also the deletion of *FgABC1* analogously affected the transcript levels of *FgABC4*. Conversely, such mutants were similar to the wild type with respect to fungicide sensitivity. Therefore, the reduction in triazole tolerance seen in the mutants of *FgABC3* is mainly resulting from the deletion of that gene. Another explanation why similar paralogs were not able to complement adequately the deletions of *FgABC3* and *FgABC4* could be the existence of distinct post-transcriptional regulation. Alternatively, the transporters missing in the deletion mutants could have distinct substrate specificities that only poorly matched those of other transporters.

Typically, fungal ABC transporters known to mediate fungicide resistance belong to the PDR (ABC-G) and the MDR (ABC-B) and to a lesser extent to the MRP (ABC-C) subfamilies. PDR transporters, whose contribution to azole resistance had been analysed in detail, are for example CDR1 and CDR2 in *Candida albicans* and PDR5 in *Saccharomyces cerevisiae* [27]. Like FgABC3, these proteins belong to group I in the ABC-G subfamily [23]. In contrast to human pathogens, far fewer functional genetic analyses have been performed for PDR transporters potentially mediating azole resistance in plant pathogenic fungi. Disruption mutants of *PMR1* (ABC-G group I) in *Penicillium digitatum*, a pathogen of citrus, exhibited increased sensitivity to azoles [28]. By employing gene replacement and overexpression in *Botrytis cinerea*, a necrotroph with a wide host range, BcAtrD (ABC-G group I) was shown to mediate azole resistance [29]. *ShAtrD*, a homolog of *BcAtrD*, was found to be overexpressed in azole resistant field isolates of *Sclerotinia homoeocarpa* causing dollar spot disease of turf grasses [30]. In *Mycosphaerella graminicola*, a pathogen infecting leaves of wheat, several ABC transporters were functionally analysed [31]. Heterologous expression of *MgAtr1* (ABC-G group III), *MgAtr2* (ABC-G group I) and *MgAtr4* (ABC-G group I) in a *S. cerevisiae* mutant with deletions of six ABC transporter genes resulted in increased tolerance against azoles. However, when the genes *MgAtr1* to *MgAtr5* were deleted individually in *M. graminicola*, no change in azole sensitivity was observed, possibly because of redundant substrate specificities [31]. At the moment, it is uncertain whether FgABC3 and FgABC4 possess very distinct substrate specificities that do not overlap sufficiently with those of other ABC transporters to allow for a compensation of the observed fungicide phenotype.

### Effects of Deletions on Virulence and Mycotoxin Production

Interestingly, in addition to fungicide tolerance, the deletion of *FgABC3* reduced the virulence on wheat and barley heads but also maize stems, suggesting an important role of the encoded protein, and thus of the molecules transported by it, during pathogenesis. The biological roles of several transporters in the PDR subfamily have been studied earlier. This applies for *FgABC3* that was previously identified in a microarray analysis as a down-regulated gene (*FgZRA1*) in a deletion mutant of *FgZEB2* (FGSG_02398) [32]. *FgZEB2* encodes a transcription factor regulating the gene cluster for zearalenone biosynthesis [33]. Deletion mutants of *FgZRA1* ( = *FgABC3* = FGSG_04580) accumulated less ZEN in liquid medium as well as in the mycelium [32]. The authors discussed that FgZRA1 is unlikely to export ZEN. The effect of *FgZRA1* deletion on ZEN production could not be explained, its role in fungicide sensitivity and virulence was not investigated. Other reports indicated that deletion mutants for the genes involved in ZEN biosynthesis were causing the same levels of FHB on wheat and barley as the wild type strains, suggesting that ZEN is dispensable for virulence on these hosts [33], [34]. In contrast to that earlier study on *FgZRA1* [32], our analysis did not show reduced ZEN levels in the deletion mutants of *FgABC3* in any of the two backgrounds studied. Differences in the genetic backgrounds and/or culture conditions between the two studies may account for this discrepancy. On the other hand, an involvement of PDR subfamily transporters in pathogenesis was demonstrated in several cases. MgAtr4 of *M. graminicola* is needed to attain full virulence on wheat and it was proposed that it may protect the pathogen against host defence molecules [35]. Similarly, BcAtrB (ABC-G group V), was described to protect *B. cinerea* against the phytoalexins resveratrol in grapevine [36] and camalexin in *Arabidopsis thaliana* [37]. In *Magnaporthe oryzae*, a hemibiotrophic pathogen of rice, the most similar protein to FgABC3 is MoABC1 (ABC-G group I). The deletion of *MoABC1* yielded mutants that were severely reduced in virulence [38]. Again, it was suggested that MoABC1 might protect the invading fungus from plant defence molecules. Later research detected a subclade within the ABC-G subfamily group I, which is distinctive to *Fusarium* spp. [39]. Functional characterisation of three members of this subclade, FcABC1 in *F. culmorum* [40], NhABC1 in *Nectria haematococca* (anamorph: *F. solani*) [39] and GpABC1 in *Gibberella pulicaris* (anamorph: *F. sambucinum*) [41] demonstrated in all cases that the encoded proteins are essential for full virulence. It was shown for the latter two transporters that they are needed to protect the pathogen from phytoalexins of their hosts, i.e. pisatin and rishitin. In conclusion, considering the literature and the results of our ZEN measurements, we propose that the biological function of FgABC3 may rather be to export a host-derived defence compound than to export the fungal secondary metabolite ZEN. Our rationale is supported by the considerably decreased levels of virulence caused by Δ*FgABC3* mutants on all three hosts tested. A virulence defect is not expected if the function of FgABC3 would be to export ZEN, because as outlined above, ZEN does not contribute to virulence. Currently, the exported molecule remains unknown, since none of the cereal metabolites that we have tested showed noteworthy variation in their effect on deletion mutants and wild type strains. Published microarray data comparing the transcriptome of *F. graminearum* during FHB on wheat and barley [42] show that *FgABC3* has the highest transcript levels among the four genes studied here (Fig. S6). In wheat, *FgABC3* transcripts peaked at 4 dpi, in barley they continuously increased until to the end of the experiment. This may indicate that FgABC3 is more important during late than early stages of infection.

Deletion mutants of *FgABC1* were impeded in infections of wheat, barley and maize irrespective of their trichothecene chemotype. The phylogenetically most similar protein to FgABC1 is FgABC4 [9], [23]; both of which are members of the MRP subfamily (ABC-C group V). Despite their similarity, deletion of *FgABC4* did not significantly affect virulence on any host tested, regardless of the chemotype. As outlined above, the opposite phenomenon was observed with respect to fungicide tolerance since the deletion of *FgABC1*, in contrast to *FgABC4*, did not cause changes compared to wild type strains, whereas deletion of *FgABC4* did so. Interrogation of published microarray data [42] indicates that transcriptional patterns of the two genes during the infection of wheat and barley were quite similar and thus inappropriate to explain why *FgABC4* cannot functionally compensate the virulence defects of the *FgABC1* deletion mutants (Fig. S6). Other explanations for this failure are contrasting post-transcriptional regulation, contrasting substrate specificities leading to discrimination between the native substrates of the two transporters or different subcellular localisations. The latter appears however questionable since the softwares Euk-mPLoc 2.0 (http://www.csbio.sjtu.edu.cn/bioinf/euk-multi-2/) and YLoc Fungi (http://abi.inf.uni-tuebingen.de/Services/YLoc/webloc.cgi) predicted for both transporters the plasma membrane as their most likely localisation (not shown).

Transporters of the MRP subfamily (ABC-C) have been less studied than those in the PDR subfamily. Recently, in *M. oryzae* three members of this subfamily have been functionally characterised [43]. Only the deletion of *MoABC5* resulted in reduced virulence on rice. The encoded protein belongs to ABC-C group V, which comprises only two members in *M. oryzae* [23]. In *Aspergillus,* several members in ABC-C group V seem to transport fungal secondary metabolites produced by nonribosomal peptide synthetase (NRPS) and polyketide synthase (PKS) enzymes [23]. *FgABC1* (FGSG_10995) resides in a supposed NRPS gene cluster [44]. In a recent microarray study, this gene and the other members of the cluster were found upregulated during infection of wounded wheat coleoptiles. Individual deletions of three genes of the cluster that encoded the transporter, an NRPS and a putative peptidoglycan deacetylase yielded mutants that exhibited reduced virulence on wheat [44]. Our Δ*FgABC1* mutants also showed strongly reduced virulence on wheat and furthermore on barley and maize. The effect on FHB in wheat was reminiscent, even though less severe, than that seen in Δ*FgTri5* mutants, which are unable to produce B-trichothecenes [45]. The latter were reported to remain restricted just to the initially infected spikelet. We observed often a similar effect in the background of NRRL 13383, which, however, is less aggressive on wheat than PH-1. In NRRL 13383, the Δ*FgABC1* mutants spread at most to two additional spikelets. Our mycotoxin analyses show that the production of trichothecenes is not impeded in the Δ*FgABC1* mutants. Therefore, the hitherto unknown secondary metabolites synthesized by the NRPS cluster, to which *FgABC1* belongs, are likely required for infection of wheat, barley and maize.

We have functionally analysed the four ABC transporter genes in two genetic backgrounds, i.e. NRRL 13383 and PH-1, to assess whether the respective genomic context may influence the effect of gene deletion. Whereas deletion of *FgABC3* and *FgABC4* caused in NRRL 13383 significantly reduced tolerance to certain class I sterol biosynthesis inhibitors, this effect was somewhat less prominent in PH-1. Due to the lack of the genome sequence of NRRL 13383 it is unknown whether this strain has exactly the same set of ABC transporters as PH-1. Variations in their numbers, sequences and regulation could cause putative compensatory effects, although other reasons may apply. Nevertheless, our results show that alterations in fungicide sensitivities resulting from gene deletions may vary in their extents in different genomic contexts. In contrast, in the virulence tests we observed rather similar consequences of the deletions in NRRL 13383 and PH-1 indicating that the virulence defects observed in the Δ*FgABC1* and Δ*FgABC3* mutants do occur independently from the trichothecene chemotype, highlighting the importance of these genes for achieving full virulence on cereals.

## Supporting Information

Figure S1 **Gene deletions.** Strategy employed to generate deletion mutants of *FgABC1* to *FgABC4* (A). Results from PCR (B) and Southern hybridisations (C) document the respective genotypes.
(PDF)

Figure S2 **Growth kinetics** ***in vitro*** **.** For each deletion, two transformants of each genetic background are compared to the respective wild type strain at three temperatures on PDA medium. Boxes on the left side show results for the PH-1 and those on the right side for the NRRL 13383 background. Each data point represents the mean of four replicated cultures.
(PDF)

Figure S3 **Formation of macroconidia** ***in vitro.*** For each deletion, two transformants of each genetic background are compared to the respective wild type strain. Data shown give the average conidial densities formed in MBB medium in four replicated cultures after incubation for 7 d at 23°C. Error bars represent SE. None of the variations between the mutants and the wild type is significant. A) PH-1 background, B) NRRL 13383 background.
(PDF)

Figure S4 **Germination of macroconidia** ***in vitro.*** For each deletion, two transformants of each genetic background are compared to the respective wild type strain. Data shown give the average frequencies of germinated macroconidia on glass slides in four replicated cultures after incubation for 24 h at 23°C. Error bars represent SD. Variations between mutants and wild types are not significant. A) PH-1 background, B) NRRL 13383 background.
(PDF)

Figure S5 **Impact of SBI class I fungicides on hyphal morphology.** For each strain, cultures containing 3 ppm of prothioconazole or fenarimol or no fungicide were grown for 4 d in liquid PDA. Only Δ*FgABC3* and Δ*FgABC4* mutants are shown, since Δ*FgABC1* and Δ*FgABC2* mutants were like the wild type references. Observation by bright field microscopy at 400x magnification.
(PDF)

Figure S6 **Transcript levels during FHB.** Data for *FgABC1* to *FgABC4* transcript levels were taken from published work (Lysoe et al., 2011). A) Time course of infection of wheat, B) of barley.
(PDF)

Table S1 **Oligonucleotides.** Used for the generation of deletion constructs, Southern blots, analytical PCR and RT-qPCR.
(XLSX)
