# Supplementary material for: Identification of ABC Transporter Genes of Fusarium graminearum with Roles in Azole Tolerance and/or Virulence
Source: PLoS One. 2013 Nov 11;8(11):e79042. doi: 10.1371/journal.pone.0079042 (PMC3823976; doi:10.1371/journal.pone.0079042)
Supplement: Figure S1 — Gene deletions. Strategy employed to generate deletion mutants of FgABC1 to FgABC4 (A). Results from PCR (B) and Southern hybridisations (C) document the respective genotypes. (PDF) [file pone.0079042.s001.pdf]

Supplemental Fig. 1

<u>wild type locus</u>
gene: ***FgABC1***
annotation: FGSG_10995
FGDB: related to multidrug resistance protein
ORF: 4632 bp
protein: 1472 aa

<u>deletion construct</u>
left flank: 1190 bp
hph cassette: 2095 bp
right flank: 1205 bp

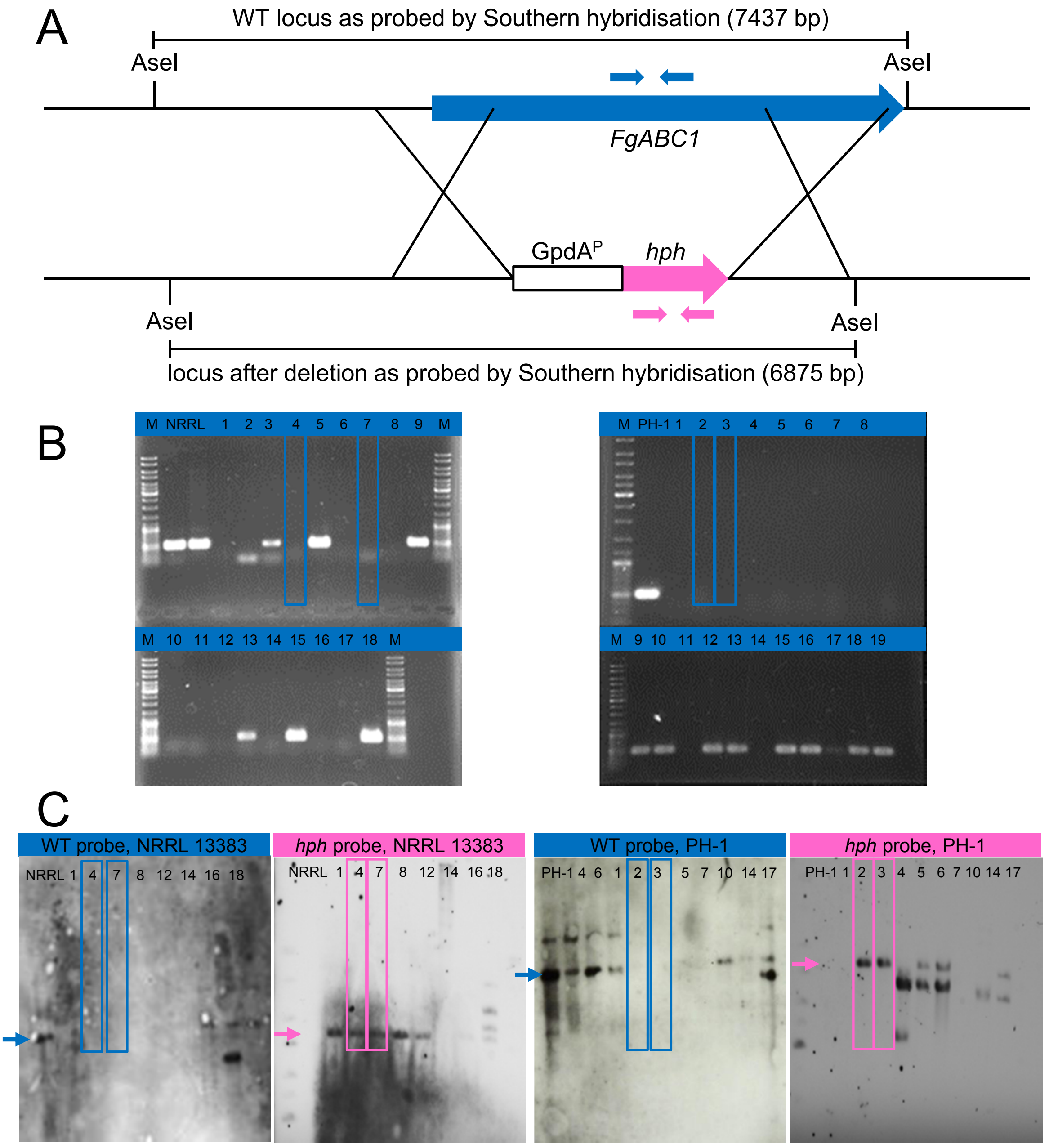

A) Strategy to delete *FgABC1*. Blue arrows give the positions of primers used to detect the wild type locus by PCR. Pink arrows indicate the respective primers for the hygromycin resistance marker (*hph*). The same primers were used to generate DIG-labelled probes used for Southern hybridisation. B) Results of PCR targeting the wild type locus. C) Results of Southern hybridisation detecting the wild type locus (blue) and *hph* (pink). Arrows indicate the expected band. Boxed lanes indicate clones used for subsequent analyses.

<u>wild type locus</u>
gene: ***FgABC2***
annotation: FGSG_17046 (formerly FGSG_08373)
FGDB: related to ABC transporter
ORF: 4836 bp
protein: 1611 aa

<u>deletion construct</u>
left flank: 1217 bp
nat1 cassette:1473 bp
right flank: 1143 bp

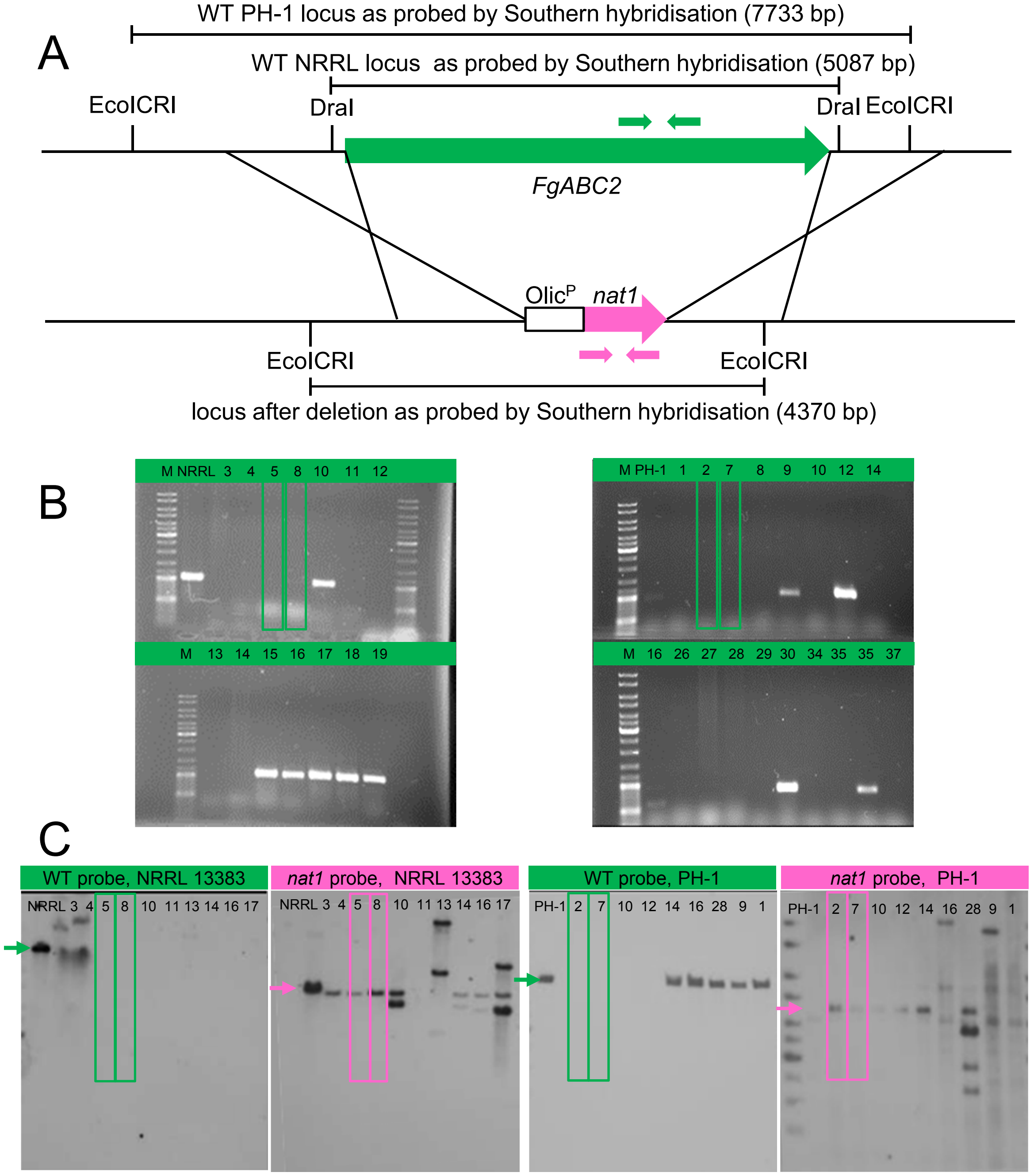

A) Strategy to delete *FgABC2*. Green arrows give the positions of primers used to detect the wild type locus by PCR. Pink arrows indicate the respective primers for the nourseothricin resistance marker (*nat1*). The same primers were used to generate the DIG-labelled probes used for Southern hybridisation. B) Results of PCR targeting the wild type locus. C) Results of Southern hybridisation detecting the wild type locus (green) and *nat1* (pink). Arrows indicate the expected band. Boxed lanes indicate clones used for subsequent analyses.

wild type locus
gene: ***FgABC3***
annotation: FGSG_04580
FGDB: probable ABC1 transport protein
ORF: 4575 bp
protein: 1489 aa

deletion construct
left flank: 896 bp
npt cassette: 1251 bp
right flank: 571 bp

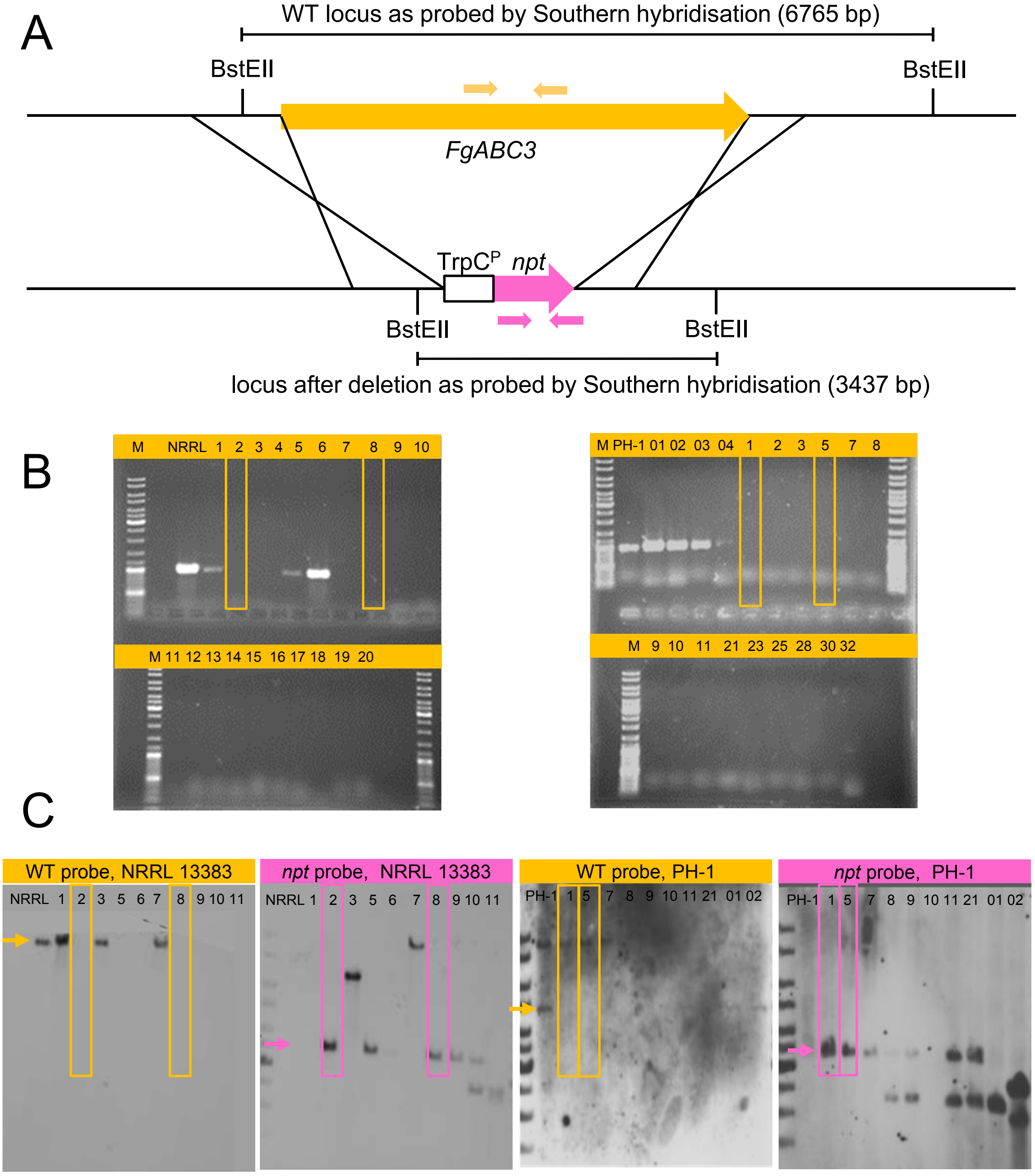

A) Strategy to delete *FgABC3*. Orange arrows give the positions of primers used to detect the wild type locus by PCR. Pink arrows indicate the respective primers for the neomycin resistance marker (*npt*). The same primers were used to generate the DIG-labelled probes used for Southern hybridisation. B) Results of PCR targeting the wild type locus. C) Results of Southern hybridisation detecting the wild type locus (orange) and *npt* (pink). Arrows indicate the expected band. Boxed lanes indicate clones used for subsequent analyses.

wild type locus
gene: ***FgABC4***
annotation: FGSG_17058 (formerly FGSG_08308)
FGDB: related to multidrug resistance protein
ORF: 4562 bp
protein: 1449 aa

deletion construct
left flank: 1026 bp
nat1 cassette:1473 bp
right flank: 1070 bp

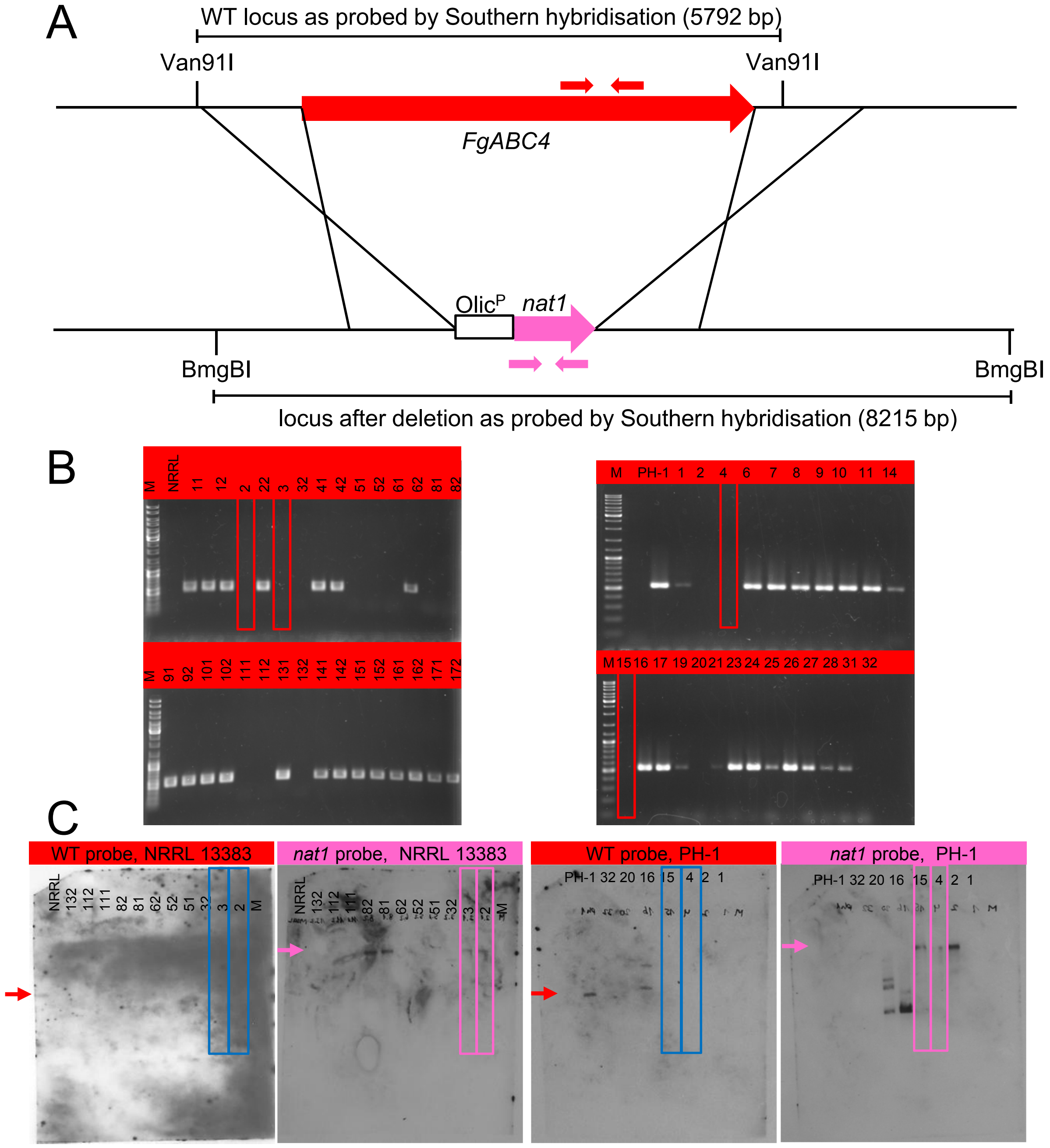

A) Strategy to delete *FgABC4*. Red arrows give the positions of primers used to detect the wild type locus by PCR. Pink arrows indicate the respective primers for the nourseothricin resistance marker (*nat1*). The same primers were used to generate the DIG-labelled probe used for Southern hybridisation. B) Results of PCR targeting the wild type locus. C) Results of Southern hybridisation detecting the wild type locus (red) and *nat1* (pink). Arrows indicate expected band. Boxed lanes indicate clones used for subsequent analyses.
