# Supplementary figures and images for: Identification of ABC Transporter Genes of Fusarium graminearum with Roles in Azole Tolerance and/or Virulence
Source: PLoS One. 2013 Nov 11;8(11):e79042. doi: 10.1371/journal.pone.0079042 (PMC3823976; doi:10.1371/journal.pone.0079042)

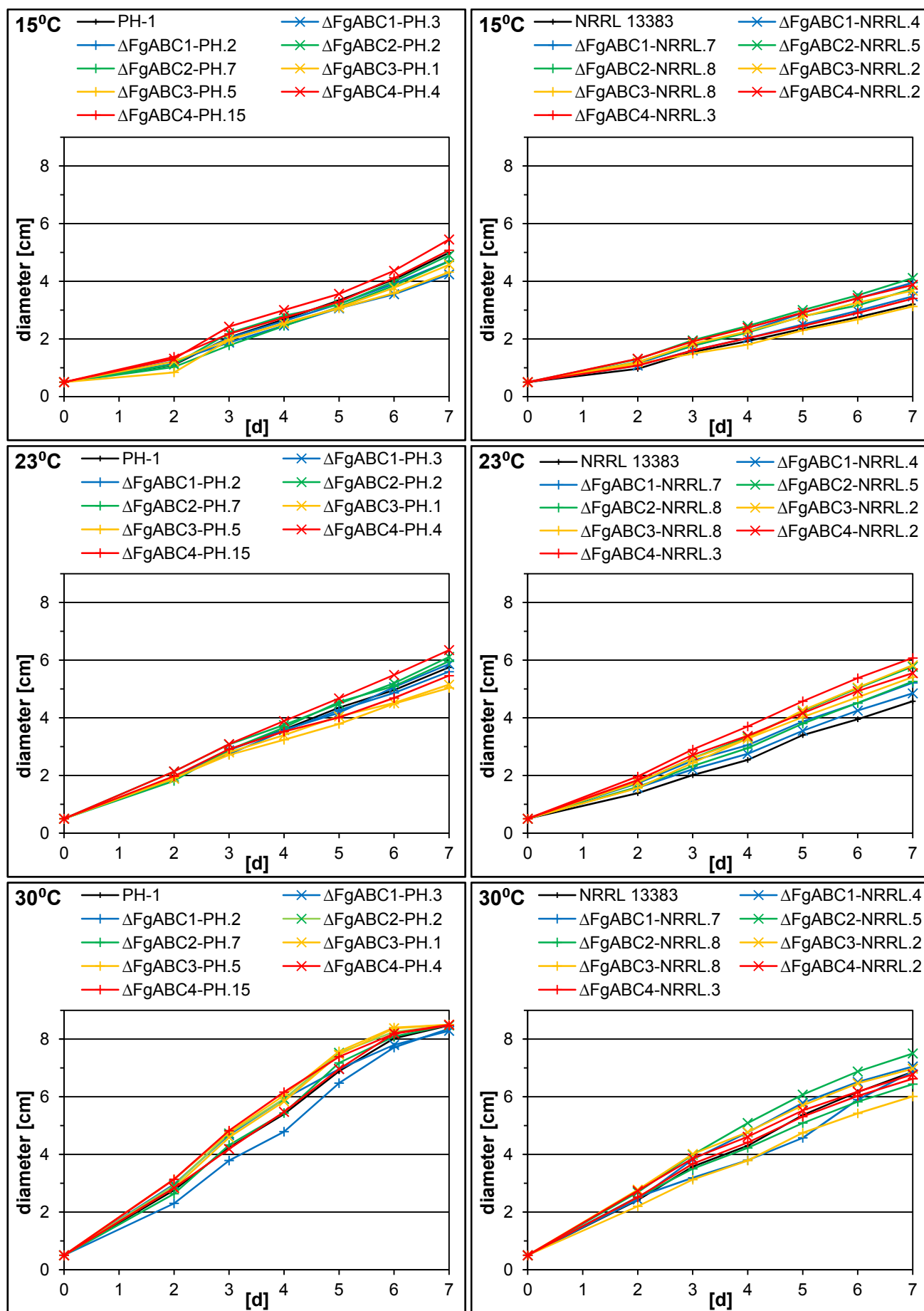

**Supplemental Fig. 2**

Supplement: Figure S2 — Growth kinetics in vitro . For each deletion, two transformants of each genetic background are compared to the respective wild type strain at three temperatures on PDA medium. Boxes on the left side show results for the PH-1 and those on the right side for the NRRL 13383 background. Each data point represents the mean of four replicated cultures. (PDF) [file pone.0079042.s002.pdf]

**A**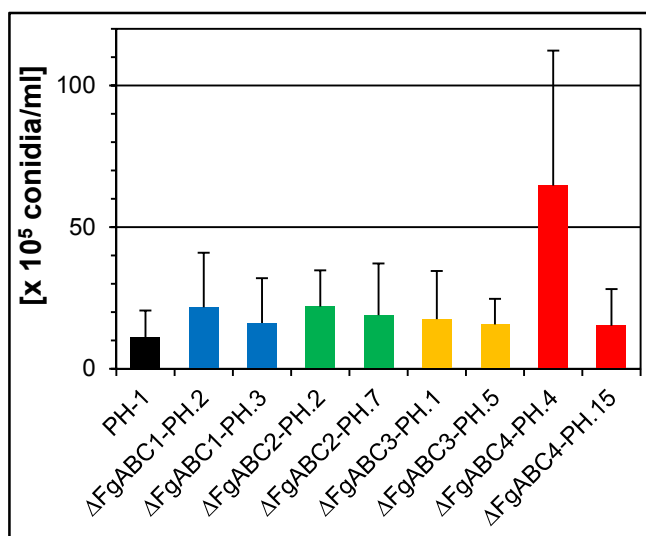**B**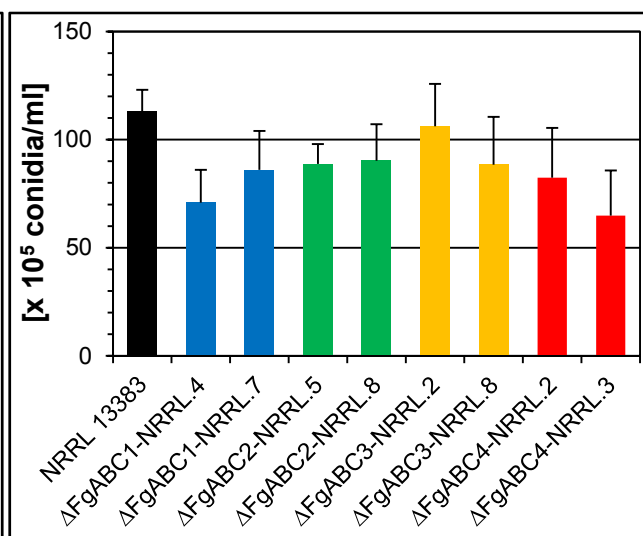**Supplemental Figure 3**

Supplement: Figure S3 — Formation of macroconidia in vitro. For each deletion, two transformants of each genetic background are compared to the respective wild type strain. Data shown give the average conidial densities formed in MBB medium in four replicated cultures after incubation for 7 d at 23°C. Error bars represent SE. None of the variations between the mutants and the wild type is significant. A) PH-1 background, B) NRRL 13383 background. (PDF) [file pone.0079042.s003.pdf]

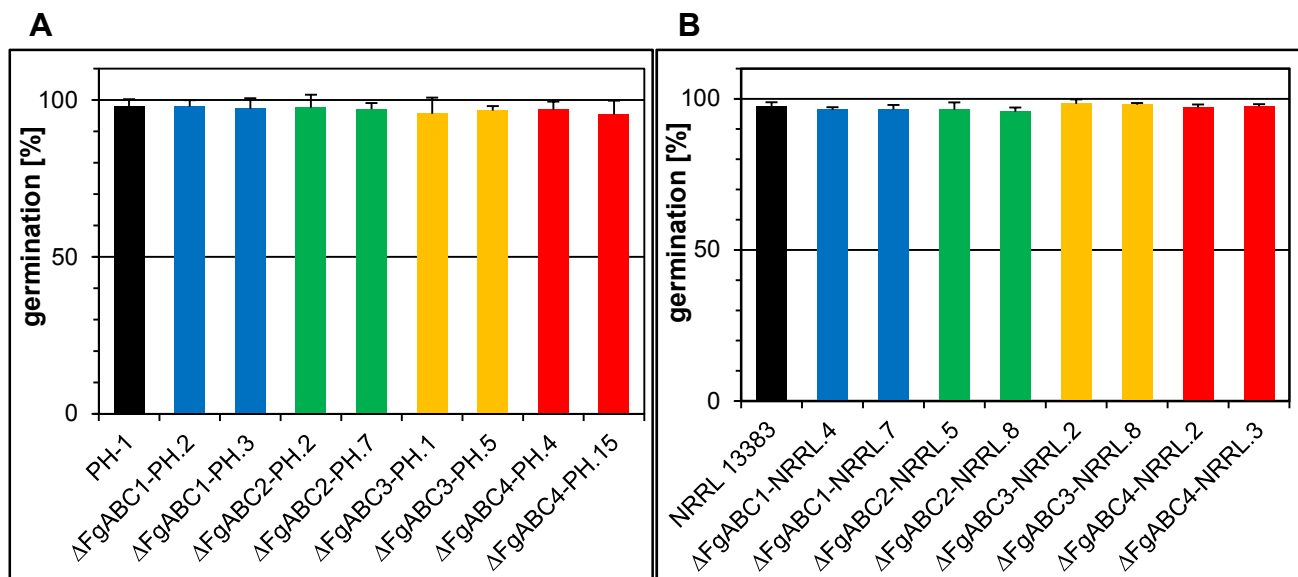

**Supplemental Figure 4**

Supplement: Figure S4 — Germination of macroconidia in vitro. For each deletion, two transformants of each genetic background are compared to the respective wild type strain. Data shown give the average frequencies of germinated macroconidia on glass slides in four replicated cultures after incubation for 24 h at 23°C. Error bars represent SD. Variations between mutants and wild types are not significant. A) PH-1 background, B) NRRL 13383 background. (PDF) [file pone.0079042.s004.pdf]

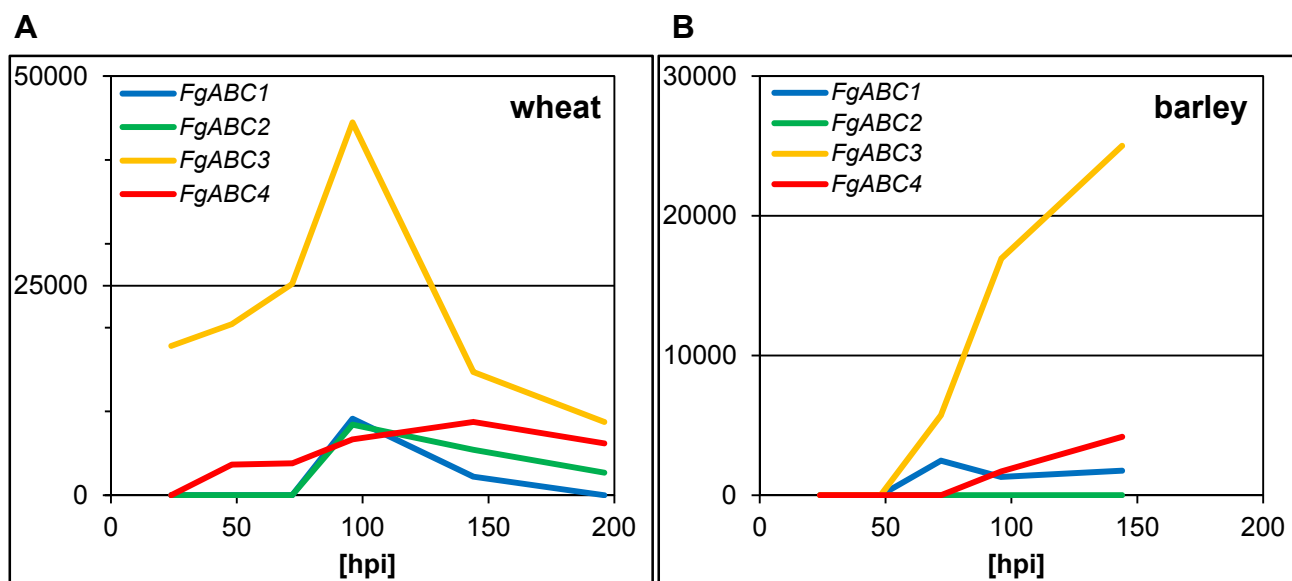

**Supplemental Figure 6**

Supplement: Figure S6 — Transcript levels during FHB. Data for FgABC1 to FgABC4 transcript levels were taken from published work (Lysoe et al., 2011). A) Time course of infection of wheat, B) of barley. (PDF) [file pone.0079042.s006.pdf]
