# Supplementary material for: Identification of ABC Transporter Genes of Fusarium graminearum with Roles in Azole Tolerance and/or Virulence
Source: PLoS One. 2013 Nov 11;8(11):e79042. doi: 10.1371/journal.pone.0079042 (PMC3823976; doi:10.1371/journal.pone.0079042)

Supplemental Figure 5

PH-1

control

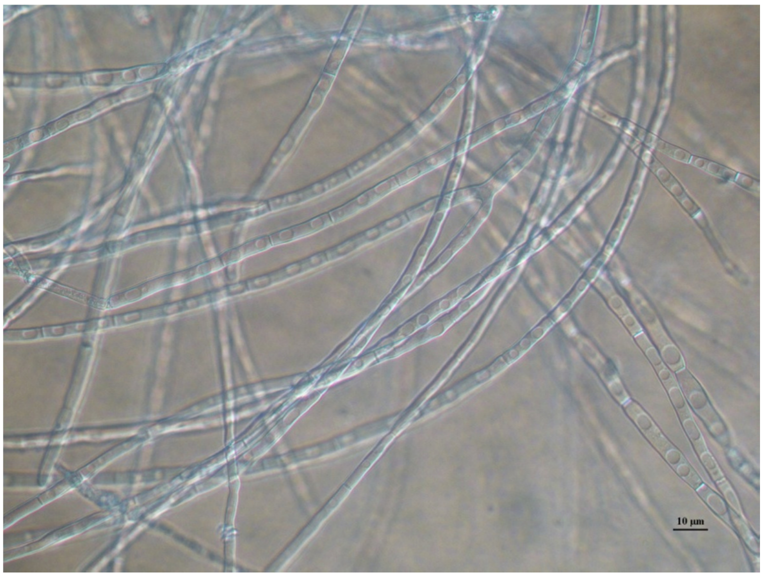

prothioconazole

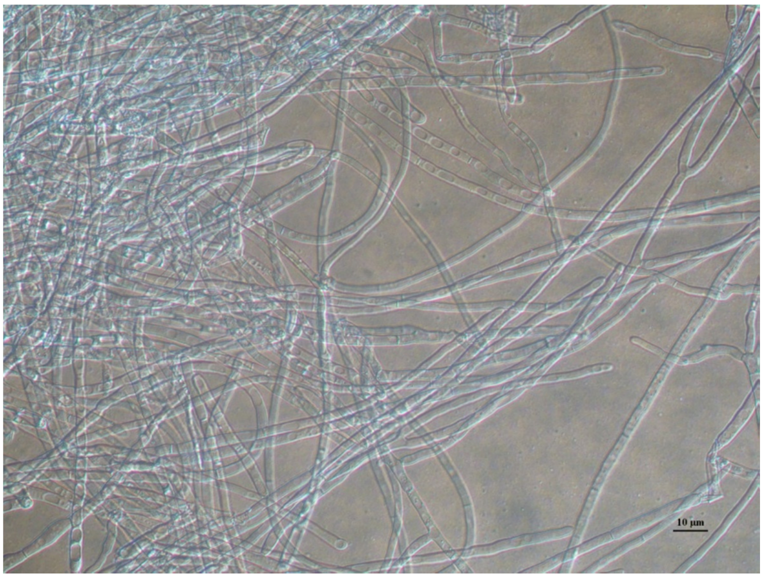

fenarimol

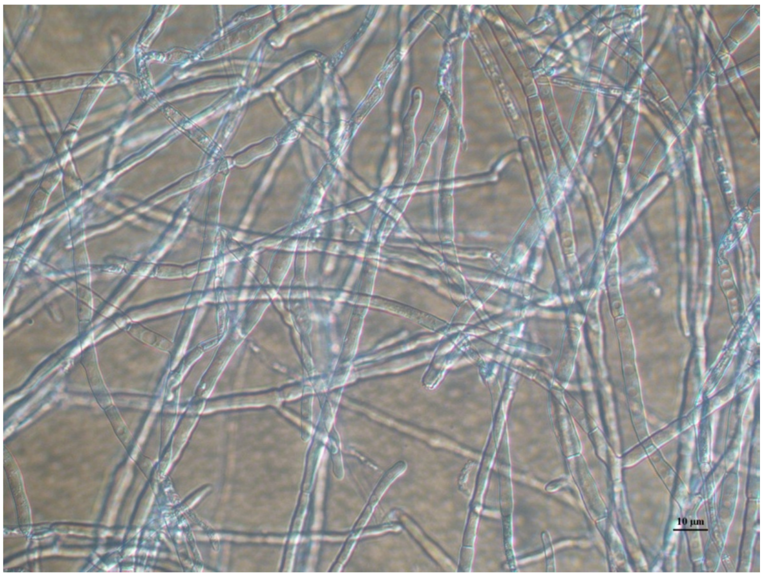

ΔFgABC3-PH.1

control

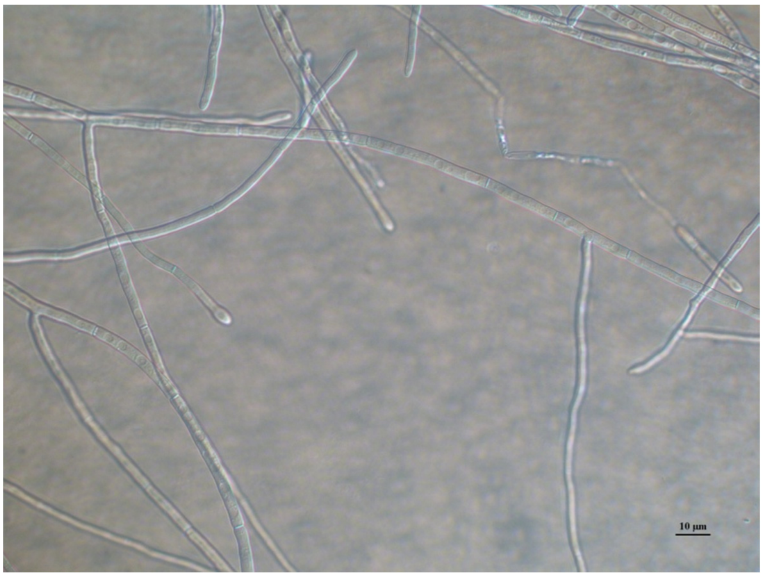

prothioconazole

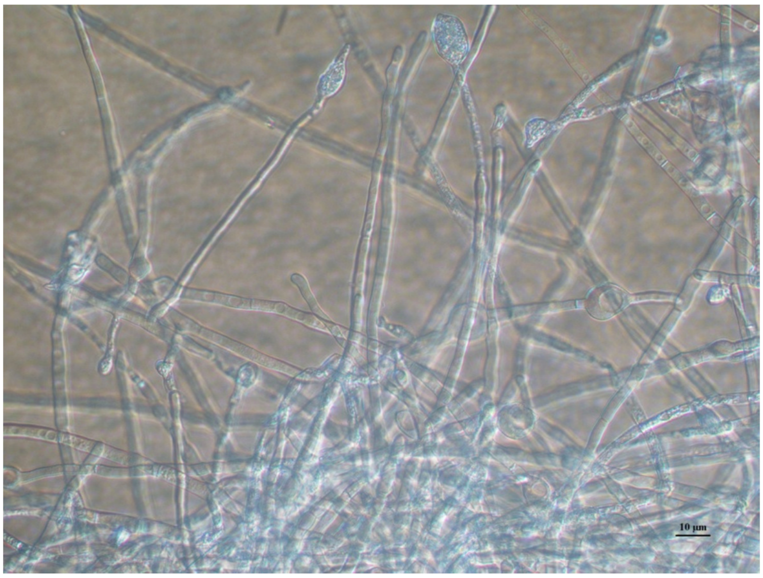

fenarimol

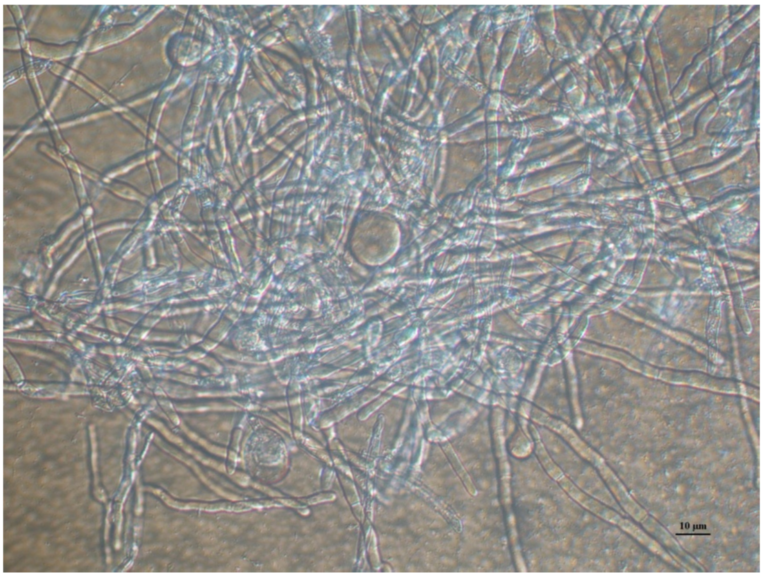

∆FgABC3-PH.5

control

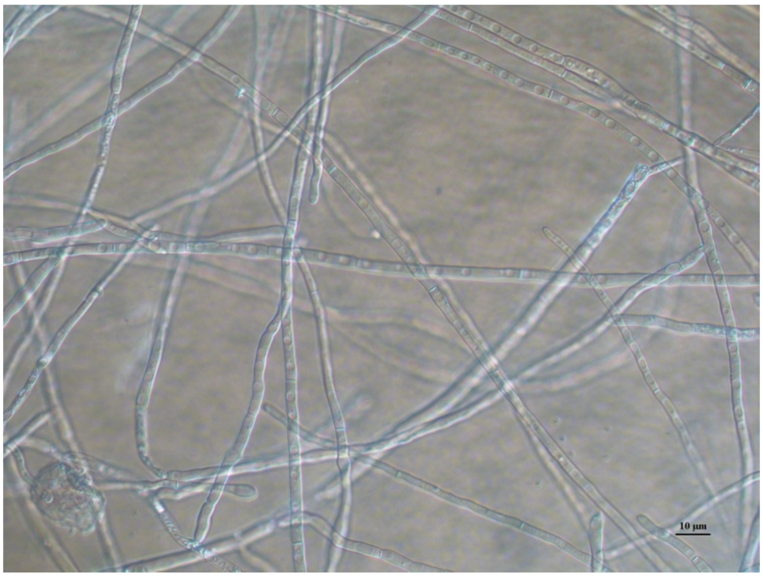

prothioconazole

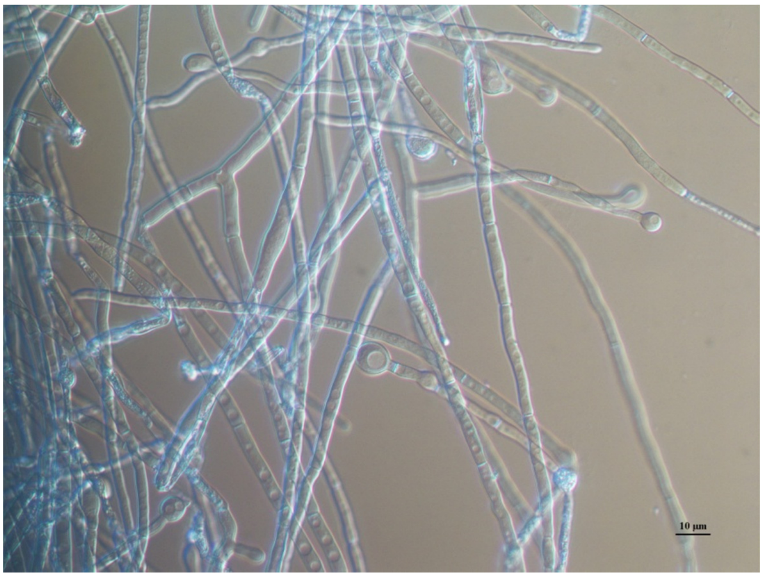

fenarimol

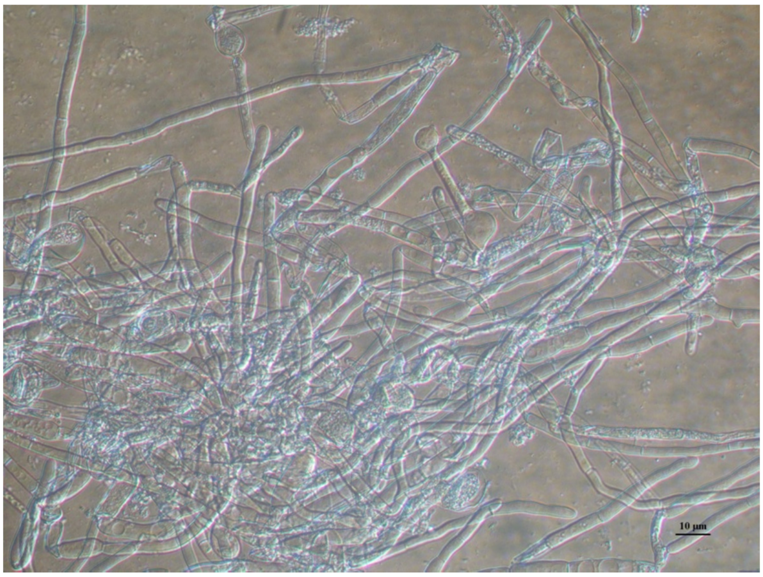

∆FgABC4-PH.4

control

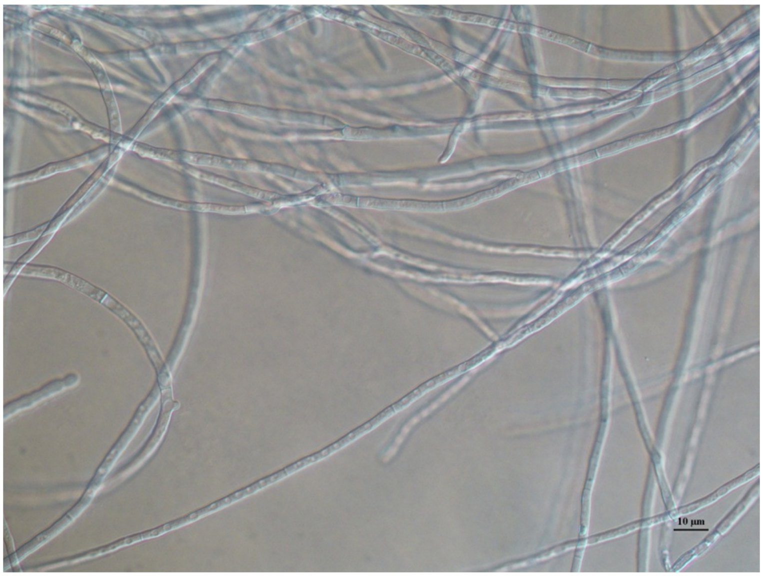

prothioconazole

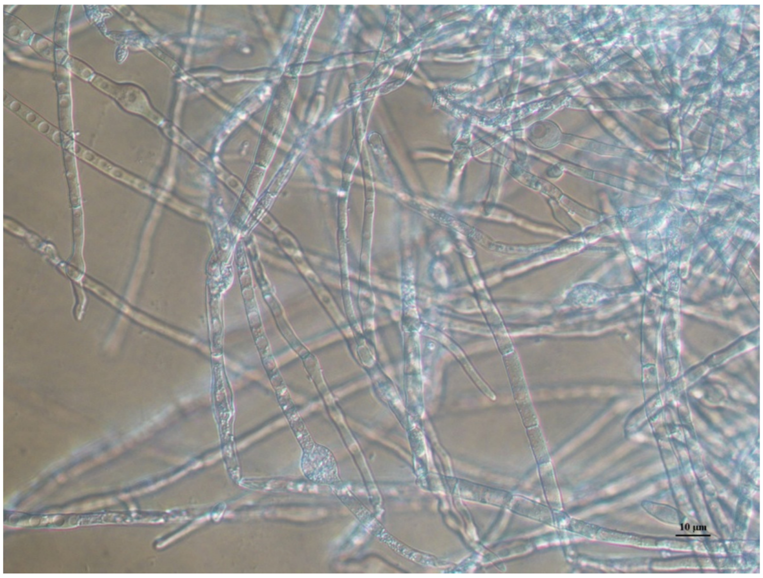

fenarimol

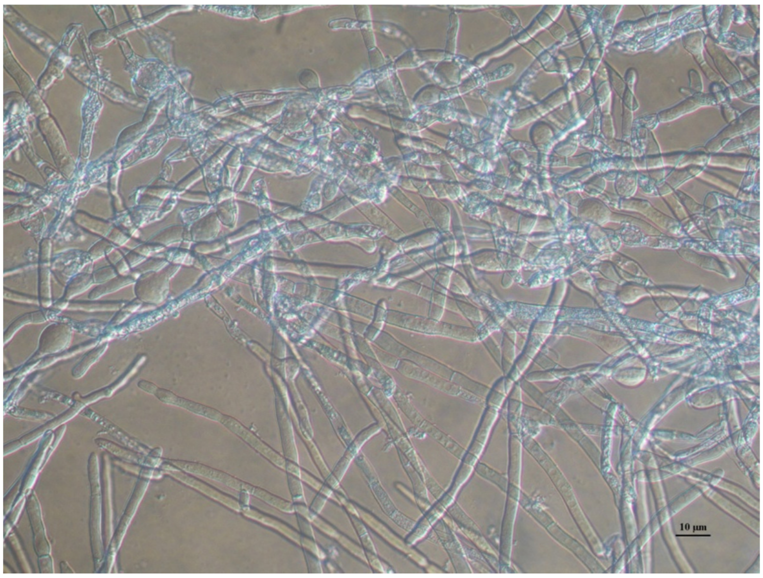

∆FgABC4-PH.15

control

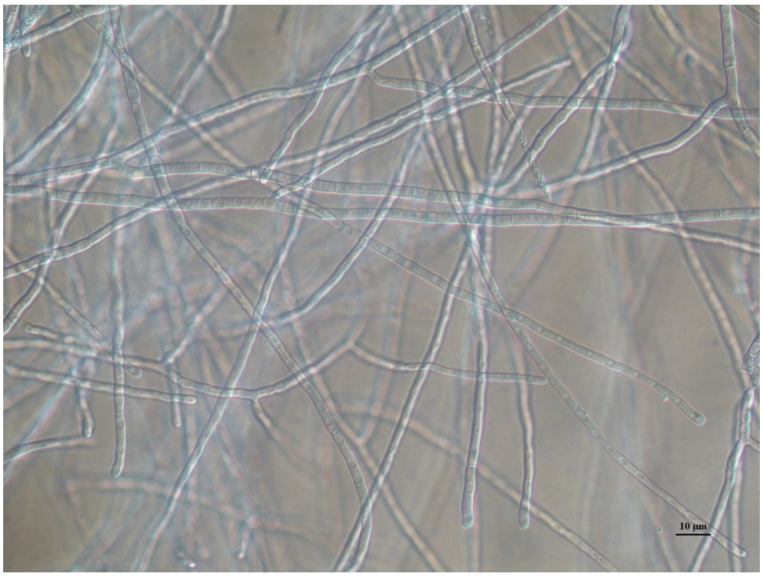

prothioconazole

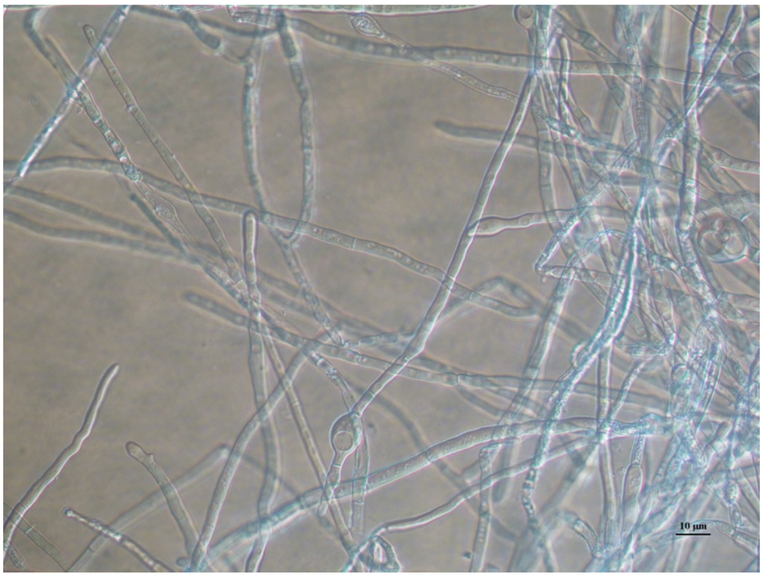

fenarimol

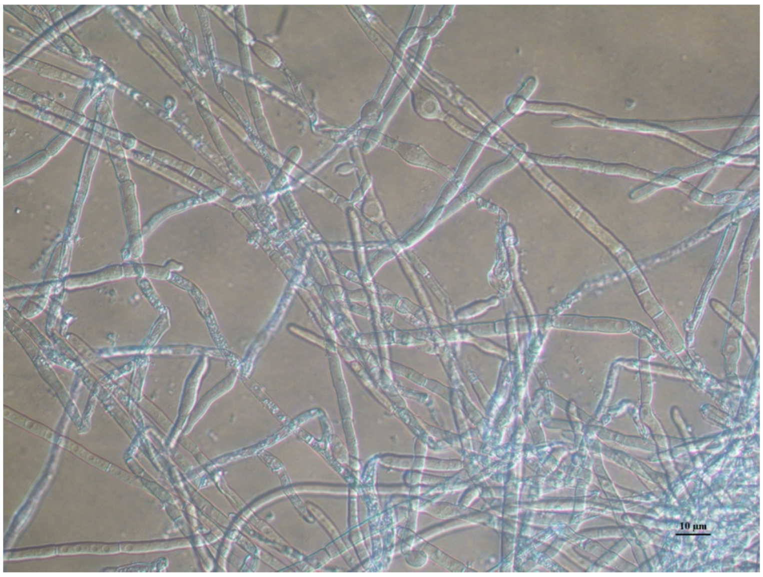

NRRL 13383

control

prothioconazole

fenarimol

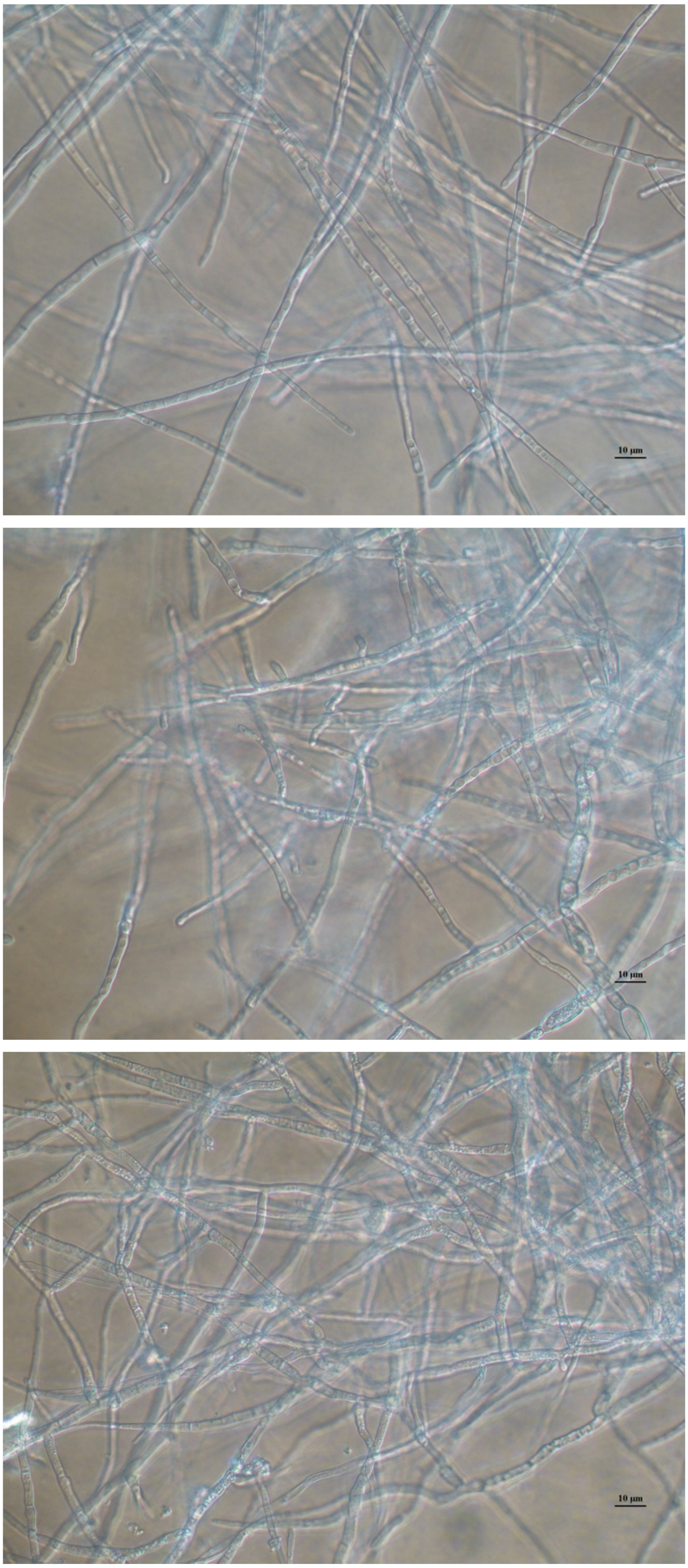

∆FgABC3-NRRL.2

control

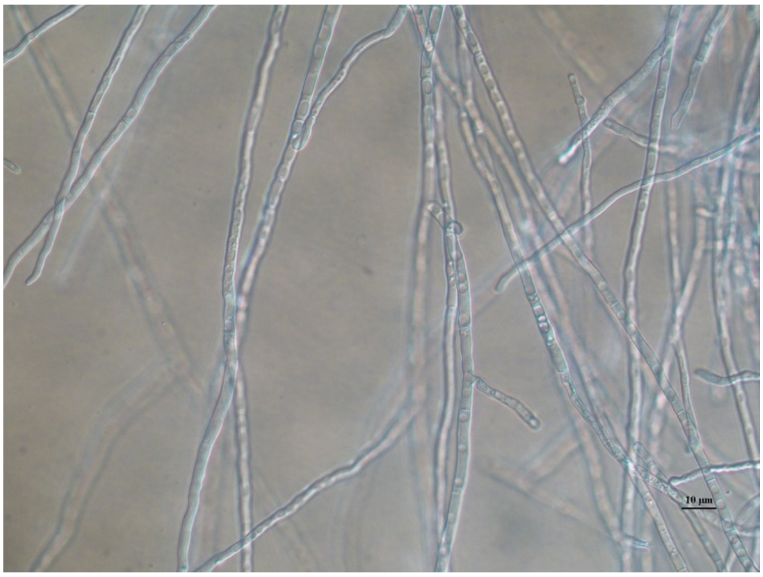

prothioconazole

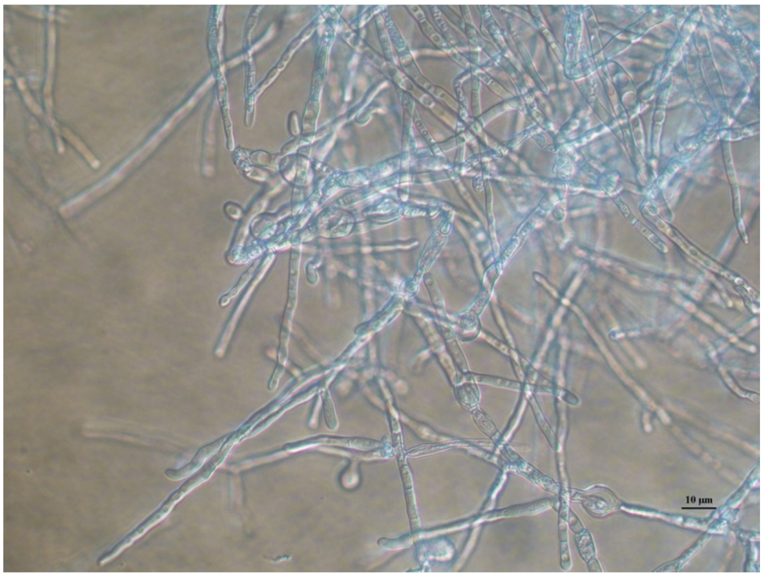

fenarimol

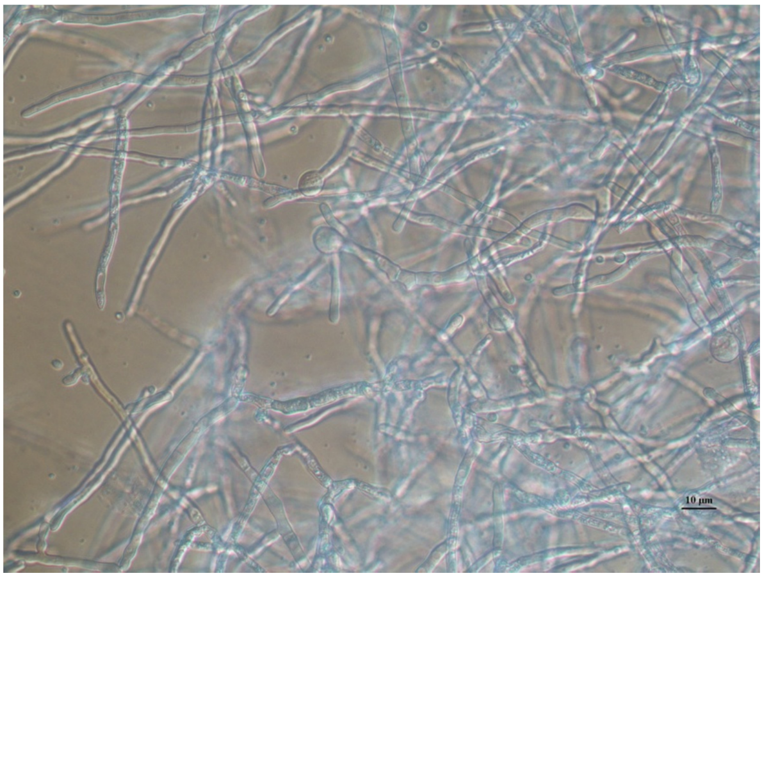

ΔFgABC3-NRRL.8

control

prothioconazole

fenarimol

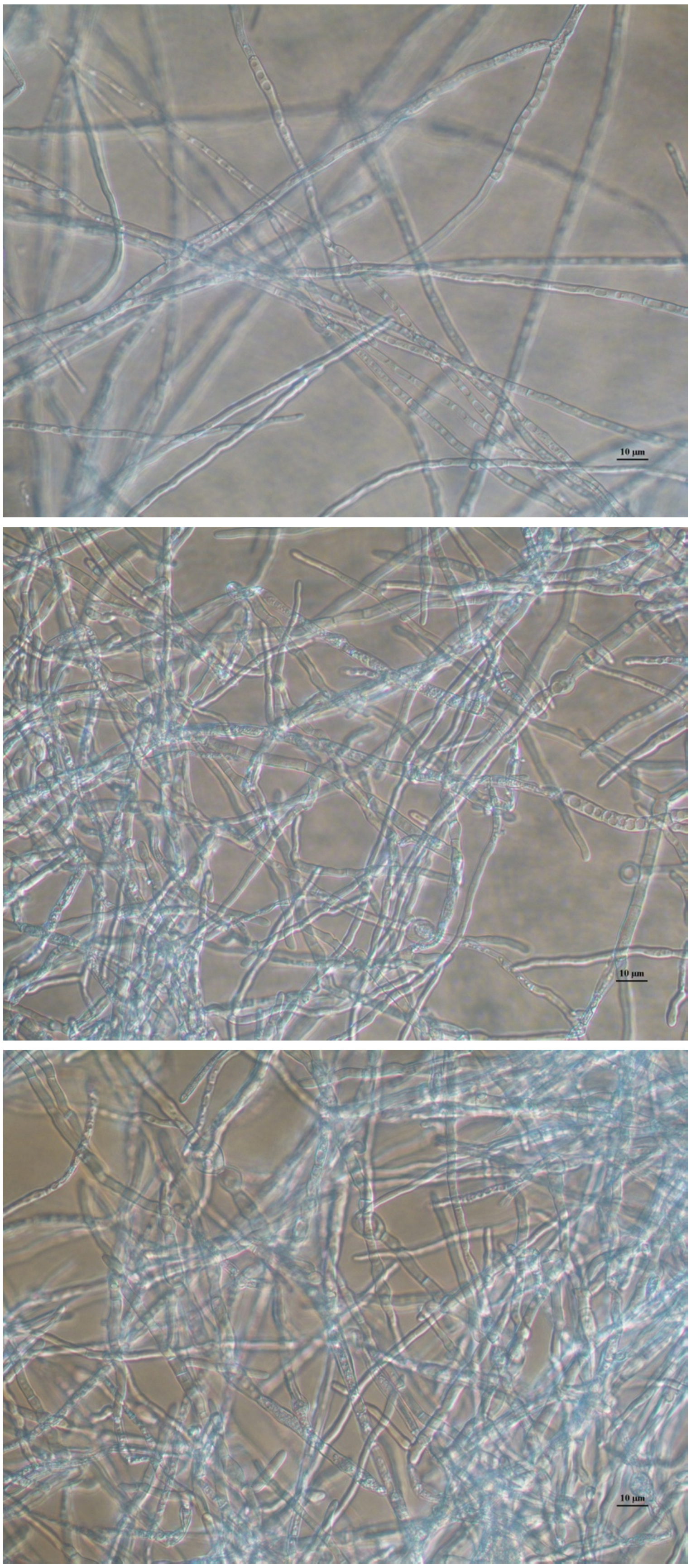

ΔFgABC4-NRRL.2

control

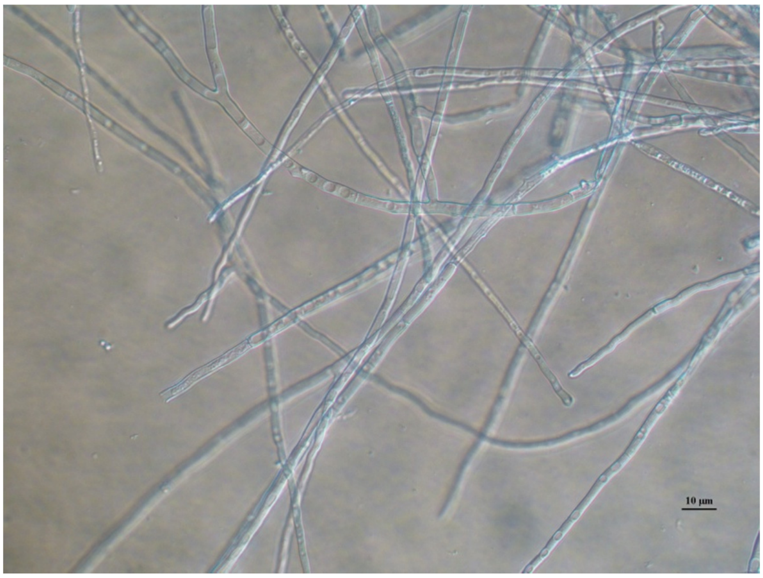

prothioconazole

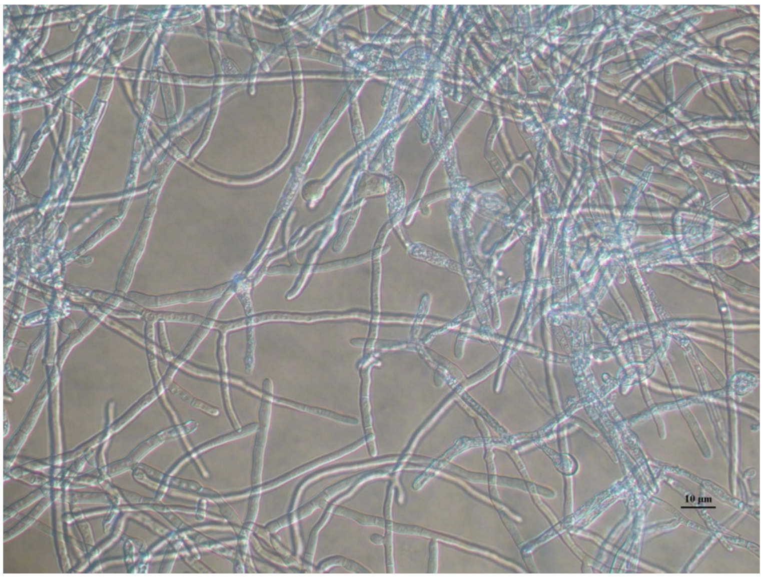

fenarimol

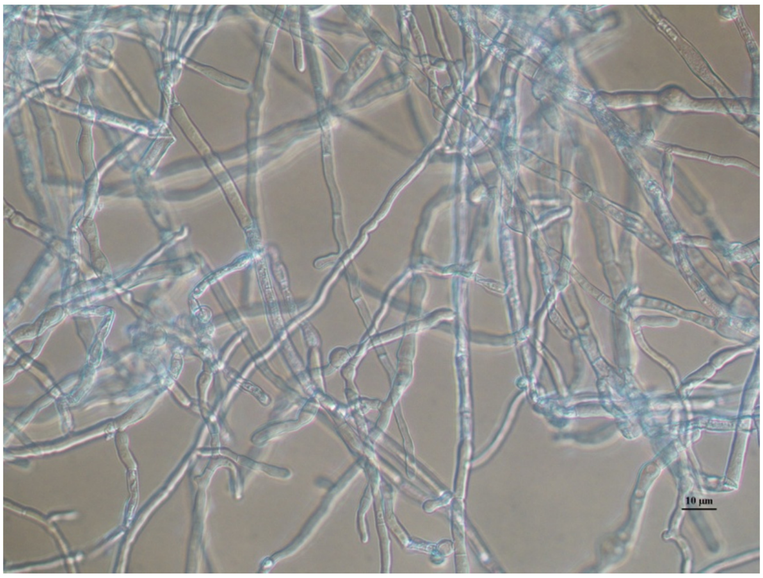

ΔFgABC4-NRRL.3

control

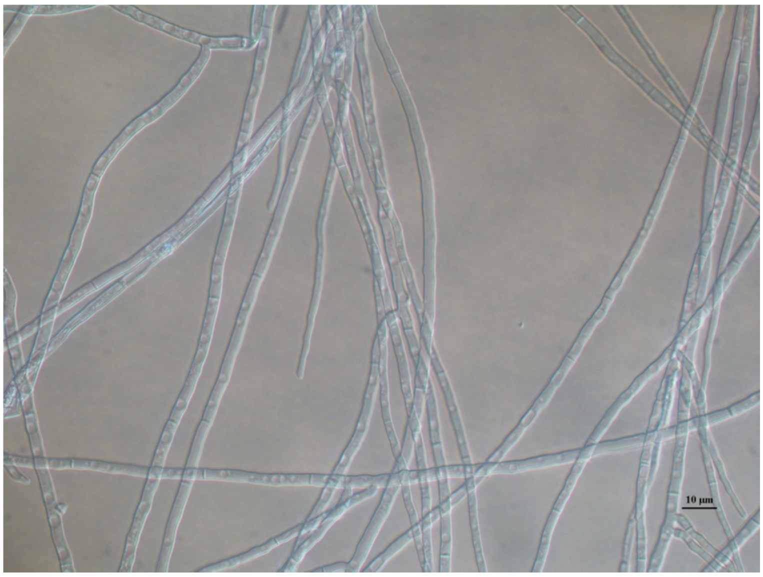

prothioconazole

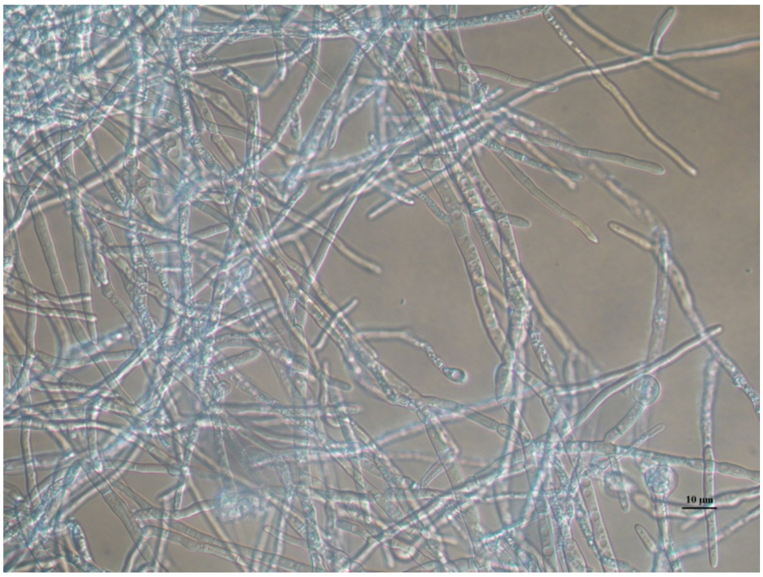

fenarimol

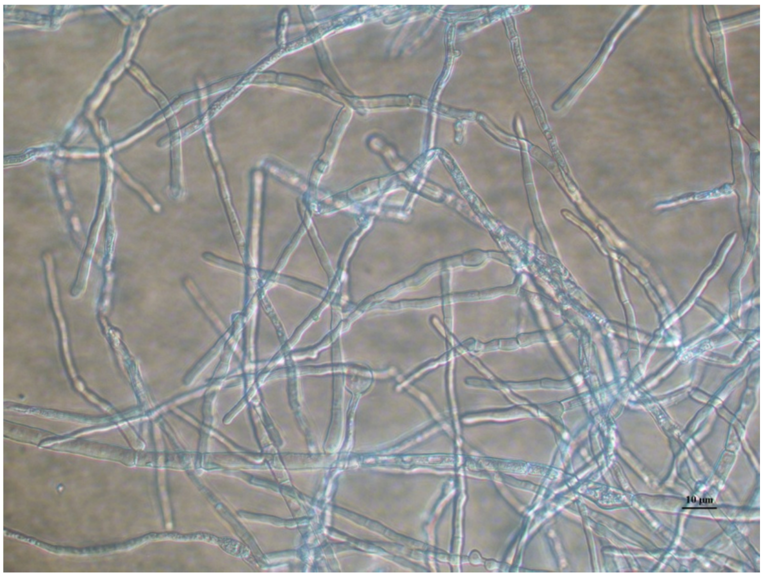

Supplement: Figure S5 — Impact of SBI class I fungicides on hyphal morphology. For each strain, cultures containing 3 ppm of prothioconazole or fenarimol or no fungicide were grown for 4 d in liquid PDA. Only ΔFgABC3 and ΔFgABC4 mutants are shown, since ΔFgABC1 and ΔFgABC2 mutants were like the wild type references. Observation by bright field microscopy at 400x magnification. (PDF) [file pone.0079042.s005.pdf]
